# Efficacy of Fibrin Sealants and Polyglycolic Acid Sheets in Reducing Postoperative Hemorrhage Following Gastric Endoscopic Submucosal Dissection: A Meta-analysis of Randomized and Observational Studies

**DOI:** 10.1007/s12029-026-01502-1

**Published:** 2026-06-05

**Authors:** Yasmin Aboelmagd, Elmoatazbellah Nasr, Mohamed Abosheisha, Ahmad Asaad, Raghunath Prabhu, Muhammed Kandeel, Mustafa Alqasem, Maan Sarsam, Ghaith Kortobi, Suhas Prasanna, Mohamed Wahb, Ahmed Swealem, Abdulrahman Ismaiel, Khaled Noureldin, Mohamed Ismaiel

**Affiliations:** 1 Internal Medicine and Clinical Haematology Department, Ain Shams University University Hospitals, Cairo, Egypt; 2 General Surgery Department, Calderdale and Huddersfield NHS Foundation Trust, Huddersfield, UK; 3 General Surgery Department, Wirral University Teaching Hospital NHS Foundation Trust, Wirral, UK; 4 General Surgery Department, Mersey and West Lancashire Teaching hospitals NHS Foundation Trust, Southport, UK; 5 General Surgery Department, Leicester Royal Infirmary, University Hospitals of Leicester NHS Foundation Trust, Leicester, UK; 6 General Surgery Department, Northumbria NHS Foundation Trust, Newcastle upon Tyne, UK; 7 General Surgery Department, University Hospitals Bristol and Weston NHS Foundation Trust, Bristol, UK; 8 Trauma and Orthopedics Department, Wexham Park Hospital, Frimley Health NHS Foundation Trust, London, UK; 9 Trauma and Orthopedics Department, Buckinghamshire Healthcare NHS Trust, Buckinghamshire, UK; 10 Department of Internal Medicine, “Iuliu Hatieganu” University of Medicine and Pharmacy, Cluj-Napoca, Romania; 11 General Surgery Department, Cairo University Hospital, Cairo, Egypt; 12 General Surgery Department, University Hospital Limerick, Limerick, Ireland; 13 Royal College of Surgeons in Ireland, Dublin, Ireland

**Keywords:** Fibrin glue, Polyglycolic acid sheets, Endoscopic submucosal dissection, Postoperative hemorrhage

## Abstract

**Background:**

Endoscopic submucosal dissection (ESD) is the standard treatment for early gastric neoplasms with low lymph node metastasis risk but is associated with notable bleeding complications, especially in high-risk patients. This study aims to evaluate the effectiveness of fibrin glue (FG) based strategies, with or without polyglycolic acid (PGA) sheets, in reducing postoperative hemorrhage.

**Methods:**

We performed a systematic search of PubMed, Scopus, and the Web of Science from inception to December 2025. We included randomized controlled trials and observational studies comparing FG or FG plus PGA sheets versus standard care in adult gastric ESD patients. The primary outcome was delayed bleeding. Risk ratios (RRs) with 95% confidence intervals (CIs) were calculated using a random-effects model.

**Results:**

Thirteen studies involving an analytical cohort of 2,728 patients were included. The meta-analysis demonstrated that the intervention significantly reduced the risk of delayed bleeding (RR: 0.49; 95% CI: 0.28, 0.84; *P* = 0.01) and overall bleeding (RR: 0.52; 95% CI: 0.29, 0.91; *P* = 0.02) compared to standard care. Subgroup analysis revealed that the benefit was primarily driven by the combination of PGA sheets and FG (RR: 0.33; 95% CI: 0.17, 0.63; *P* < 0.001), while FG alone did not show a statistically significant reduction (RR: 0.62; *P* = 0.26). Similarly, symptomatic bleeding was significantly reduced only in the PGA + FG subgroup (RR: 0.25; *P* = 0.009). No significant differences were observed regarding acute bleeding (RR: 0.90; *P* = 0.81) or procedural perforation (RR: 1.09; *P* = 0.87).

**Conclusion:**

The combined application of PGA sheets and fibrin glue significantly reduces delayed hemorrhage after gastric ESD, likely due to a dual mechanism of mechanical shielding and biological stabilization. Fibrin glue alone does not consistently confer a protective benefit.

**Supplementary Information:**

The online version contains supplementary material available at 10.1007/s12029-026-01502-1.

## Introduction

Gastric cancer is the fifth most frequently diagnosed malignancy globally [1]. Endoscopic submucosal dissection (ESD), a minimally invasive technique that surpasses endoscopic mucosal resection, has become widely adopted for managing gastric epithelial neoplasms and is considered the standard approach for lesions with low lymph node metastasis risk [2–4]. Although ESD offers benefits like quicker recovery and preservation of normal gastrointestinal integrity, it is still associated with notable complications, most importantly postoperative bleeding and perforation. Post-ESD bleeding may trigger hemorrhagic shock and adversely influence patient outcomes, with incidence rates reported at 4%–8% in early gastric cancer and increasing to 21%–38% in individuals receiving antithrombotic therapy or undergoing large resections [5–7]. High-risk patients on warfarin or undergoing regular dialysis also show higher rates of delayed and recurrent bleeding [8]. Existing preventive options include post-ESD coagulation (PEC). The search, coagulation, and clipping (SCC) technique is a well-established approach, with some retrospective evidence suggesting potential benefit over PEC; however, these strategies do not completely eliminate the risk of postoperative bleeding [9, 10]. Similarly, careful coagulation of visible vessels reduces bleeding risk; however, postoperative hemorrhage still occurs, particularly in high-risk patients [11]. This underscores the importance of establishing effective measures to prevent hemorrhage following upper gastrointestinal ESD.

Fibrin glue (FG), a hemostatic and tissue-adhesive agent derived mainly from human or bovine fibrinogen and thrombin, is commonly employed in neurosurgery, ophthalmology, and other surgical fields [12, 13]. When applied to iatrogenic ulcers, it aids hemostasis by promoting clot formation and supports wound healing through tissue sealing without triggering a foreign body reaction. In ESD, FG has been used alone or alongside polyglycolic acid (PGA) sheets to reduce delayed bleeding in high-risk patients [14]. A Recent study have shown that combining PGA sheets with fibrin glue (FG) reduces post-ESD hemorrhage rates to 6.7%, compared with 22.0% in controls; notably, these findings are derived from cohorts restricted to high-risk patients (e.g., antithrombotic use or large resections), explaining the elevated control event rates [15]. Although effective, this technique requires greater procedural skill and longer operative time than standard ESD [16]. Nevertheless, comprehensive data on the efficacy of FG combined with PGA sheets in reducing postoperative hemorrhage following gastric ESD are inconclusive. This systematic review and meta-analysis aim to evaluate its effectiveness.

## Materials and Methods

### Study Design

This study was conducted as a systematic review and meta-analysis evaluating the effectiveness of fibrin glue–based strategies, with or without polyglycolic acid (PGA) sheets, in preventing delayed bleeding after gastric endoscopic submucosal dissection (ESD). The review methodology followed the PRISMA (Preferred Reporting Items for Systematic Reviews and Meta-Analyses) guidelines [17].

### Search Strategy

A comprehensive literature search was performed in PubMed, Embase, Scopus, and the Cochrane Library from database inception to December 2025. The search strategy incorporated combinations of the following keywords and their variants: “endoscopic submucosal dissection,” “gastric ESD,” “delayed bleeding,” “post-ESD bleeding,” “fibrin glue,” “fibrin sealant,” “polyglycolic acid,” and “PGA sheet.” Reference lists of relevant original studies and review articles were manually screened to identify additional eligible studies.

### Eligibility Criteria

Studies were considered eligible if they met the following criteria: (1) included adult patients undergoing gastric ESD for gastric neoplasms (adenoma, early gastric cancer, or premalignant lesions); (2) evaluated fibrin glue or fibrin sealant applied to the post-ESD ulcer, either alone or in combination with PGA sheets; (3) included a comparator group receiving standard post-ESD management without sealants or PGA sheets; and (4) reported at least one relevant bleeding-related outcome. Eligible study designs included randomized controlled trials, prospective observational studies, and retrospective comparative studies, including those using propensity score matching. Exclusion criteria comprised studies involving non-gastric ESD, benign lesions only, single-arm case series, case reports, reviews, editorials, animal studies, and conference abstracts without extractable data.

### Outcome Definitions

Outcomes were categorized into bleeding-related and clinical domains. The primary outcome was delayed bleeding, defined as clinically significant post-ESD hemorrhage occurring after the immediate post-procedural period (typically ≥ 24–48 h after ESD), and generally characterized by one or more of the following: requirement for endoscopic hemostatic intervention, need for blood transfusion, or a significant drop in hemoglobin level, according to the definitions used in the individual studies. Secondary outcomes included early bleeding, overall bleeding, incidence of perforation, and other procedure-related adverse events.

### Study Selection

After removal of duplicate records using EndNote X9, two reviewers independently screened the titles and abstracts of all retrieved studies to identify potentially eligible articles. Full-text review was subsequently performed for studies meeting the inclusion criteria. Disagreements regarding study eligibility were resolved through discussion and consensus, with adjudication by a third reviewer when necessary.

### Quality Assessment and Publication Bias

Two independent reviewers assessed the methodological quality and risk of bias of included randomized controlled trials using the Cochrane Risk of Bias 2.0 (RoB 2) tool [18], evaluating randomization, deviations from intended interventions, missing outcome data, outcome measurement, and selective reporting. Observational studies were assessed using the Newcastle–Ottawa Scale (NOS), focusing on cohort selection, comparability of study groups, and outcome assessment. Publication bias was evaluated by visual inspection of funnel plots when at least ten studies were available for a given outcome.

### Statistical Analysis

Risk ratios (RRs) with 95% confidence intervals (CIs) were calculated for dichotomous outcomes, including delayed bleeding, early bleeding, overall bleeding, reintervention, and adverse events. A random-effects model was applied for all analyses to account for anticipated clinical and methodological heterogeneity across studies. Statistical heterogeneity was assessed using the Higgins’ I² statistic, with thresholds interpreted according to Cochrane recommendations [19]. Sensitivity analyses were conducted by sequential exclusion of individual studies to assess the robustness of pooled estimates. Prespecified subgroup analyses were performed based on intervention type (fibrin glue alone vs. fibrin glue with PGA sheets). All statistical analyses were conducted using R software.

## Results

### Search Results

**Fig. 1:**
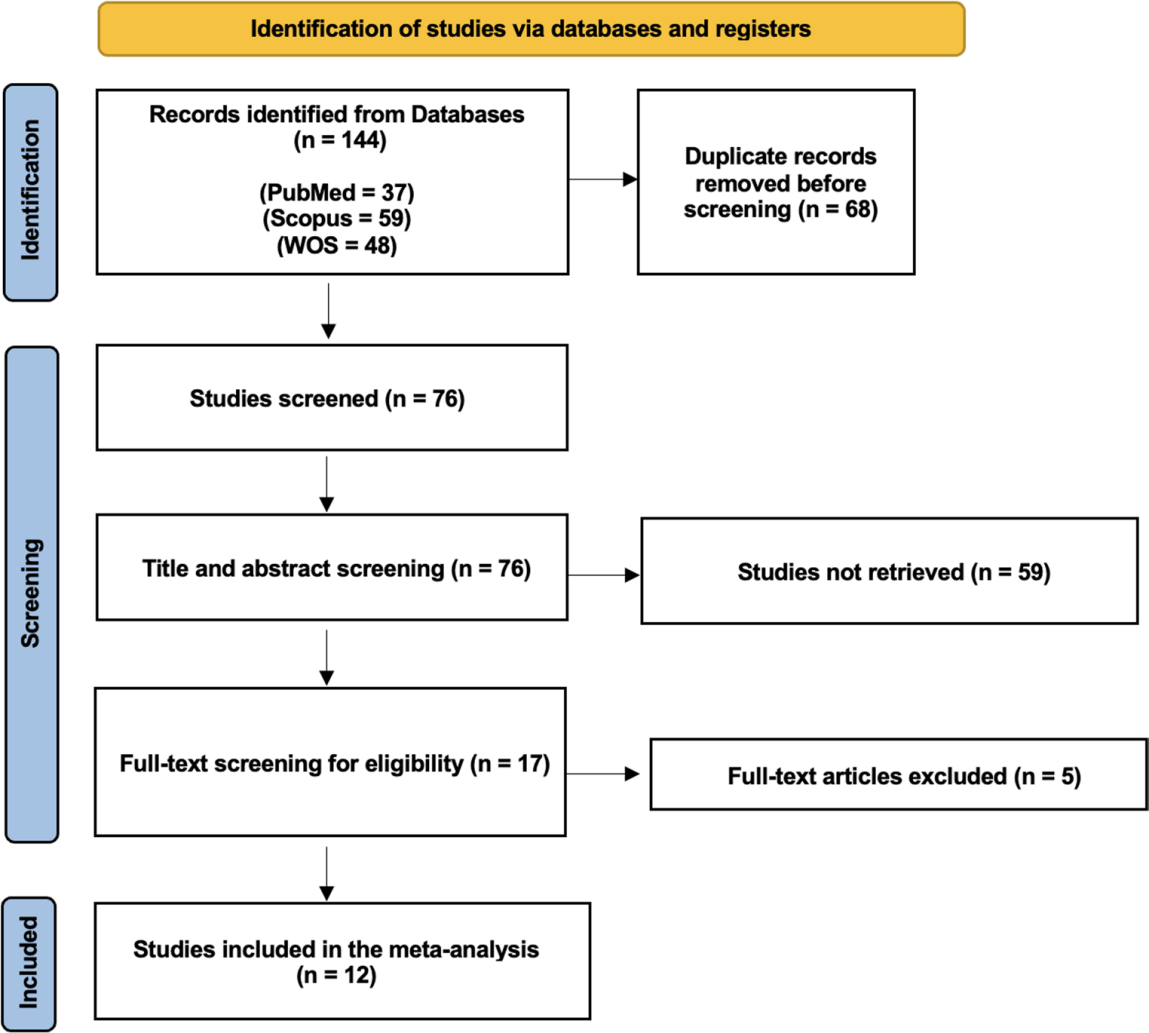
Prisma flow chart of the included studies

The literature search identified 144 records from PubMed (*n* = 37), Scopus (*n* = 59), and Web of Science (*n* = 48). After removing 68 duplicates, 76 studies were screened by title and abstract, of which 59 were excluded. Seventeen full-text articles were assessed for eligibility, and 5 were excluded, leaving 12 studies included in the meta-analysis (Fig. 1).

### Summary of the Included Studies

This meta-analysis included 13 studies comprising a total analytical cohort of 2,728 patients who underwent gastric endoscopic submucosal dissection. These studies were conducted across Japan, South Korea, and China, with publication dates spanning from 2015 to 2025. The included research employed a variety of robust methodologies, including prospective randomized controlled trials (RCTs), multicenter retrospective studies, and analyses utilizing propensity score matching (PSM) to control for baseline imbalances. Two main intervention strategies were evaluated: the application of fibrin glue or sealant alone and the combined use of polyglycolic acid (PGA) sheets fixed with fibrin glue. Most studies focused on high-risk populations, specifically those on antithrombotic therapy or with large lesions (specimen size ≥ 40 mm). Resected specimen size across the included studies generally ranged from approximately 30 to 53 mm, with most studies reporting values around or above the ≥ 40 mm threshold for large resections. Tumor size was reported in fewer studies, ranging from approximately 12.6 to 26 mm, with only one study in the fibrin only group reporting very small lesions (~ 3–4 mm) [20]; several studies did not report tumor size. Table 1 represents detailed summary of the included studies.

**Table 1:** Summary of the Included Studies

Study ID	Center	Study design	Study period	Intervention and sample size	Comparator and sample size	Follow-up duration (mean)
Abiko 2021 [21]	Single center, Japan	Retrospective case series	April 2018 to January 2020	PMSCC (PGA + fibrin glue) + MSCC (*n* = 9 lesions)	MSCC alone (*n* = 114 lesions)	30 days
Fukuda 2016 [22]	Single center, Japan	Retrospective, single-center analysis	July 2012 to December 2015	PGA sheets + Fibrin glue (*n* = 104)	Non-sealing control (*n* = 70)	12.7 days (range 1–32)
Kawata 2018 [23]	Single center, Japan	Retrospective, single-center study	April 2014 to Sept 2015	Covering (PGA+ Glue) (*n* = 52 lesions; *n* = 38 patients)	Control (*n* = 53 lesions; *n* = 46 patients)	28 days
Kikuchi 2018 [24]	Single center, Japan	Prospective, single-center study	July 2014 to Nov 2015	Autologous Glue ± PGA (*n* = 22 lesions; *n* = 20 patients)	NO comparator (Single-arm)	8 weeks
Kikuchi 2019 [25]	Single center, Japan	Prospective, single-center study	December 2014 to September 2017	PGA + Autologous Glue (*n* = 38 patients)	Conventional group (*n* = 85 patients)	8 weeks
Tsuji 2015 [26]	Single center, Japan	Non-randomized trial with historical control	Study group: July 2013 – Feb 2014; Historical: Jan 2013 – July 2013	PGA sheets + Fibrin Glue (*n* = 45 ulcers / 41 patients)	Historical Control (*n* = 41 ulcers / 37 patients)	4 weeks
Kataoka 2019 [27]	Multicenter, Japan	RCT	September 2014 to September 2016	PGA sheets + Fibrin Glue (*n* = 67)	Standard care (Control) (*n* = 70)	4 weeks
Chen 2025 [28]	Multi-center (4 centers), China	Retrospective multicenter study	March 2015 to June 2024	Fibrin sealant (*n* = 355)	Control (no fibrin sealant sprayed) (*n* = 306)	1 month
Tan 2016 [29]	Single center, China	Retrospective cohort study	January 2011 to August 2014	FG Spray (*n* = 96)	Non-FG (*n* = 301)	30 days
Wang 2020 [30]	Single center, China	Retrospective, Propensity Score Matched	January 2012 to December 2017	Combined hemostats (Fibrin sealant) (*n* = 115)	Electrocautery group (*n* = 115)	4 weeks
Kim 2025 [31]	Single center, South Korea	RCT	July 2021 to March 2023	Fibrin Glue (*n* = 126)	Control (*n* = 126)	8 weeks
Zhang 2025 [20]	Single center, China	Retrospective, Propensity Score Matched	November 2022 to November 2024	Fibrin Glue (*n* = 102)	Conventional group (*n* = 102)	4 weeks
Lee 2023 [32]	Multicenter, South Korea	RCT	July 1, 2020, to June 22, 2022	Fibrin Glue (*n* = 125)	Standard ESD (Control) (*n* = 122)	4 weeks

*FG* fibrin glue, *PGA* polyglycolic acid, *PMSCC* polyglycolic acid sheets and fibrin glue combined with the modified search, *MSCC* modified search, coagulation, *ESD*: endoscopic submucosal dissection, *RCT* randomized controlled trial

### Baseline Characteristics of the Included Studies

Patients were generally older adults, with reported mean/median ages ranging from 60.6 to 78.5 years, and most cohorts were male-predominant. Included lesions reflected the typical spectrum seen in gastric ESD populations, including adenoma/low-grade dysplasia, high-grade dysplasia, and gastric carcinoma, although the proportions varied across studies. Lesions were distributed across gastric regions (upper, middle, and lower stomach), with many studies reporting a substantial proportion in the lower stomach. Antithrombotic exposure was common and, in several studies, cohorts were entirely composed of patients receiving antithrombotic therapy. Reported agents included aspirin, clopidogrel (and other thienopyridines), cilostazol, dual antiplatelet therapy, warfarin, and DOACs, with some studies also reporting patients on multiple agents. Tables 2 and 3 represents the full baseline characteristics of the included studies.  

**Table 2:** Baseline characteristics of the included studies

Study ID	Age (Median/Mean)	Sex (M/F)	Type of Tumor (Histology)	Location (U / M / L)	Antithrombotic Use (%)
Abiko (2021) [21]	Total: 73 (IQR 69–79)	Total: 98 (81.0%)	**Adenocarcinoma** (86.2%) and **Adenoma** (11.4%).	Upper (13.0%), Middle (48.0%), and Lower (39.0%).	22.8%
Chen (2025) [28]	**I**: 66; **C**: 64 (Median)	**I**: 271 (76.3%); **C**: 221 (72.2%)	**Gastric Carcinoma** (40.5–48.5%), **LGIN** (31.3–43.8%), and **HGIN** (15.7–20.3%).	Upper (25.9–43.1%), Middle (32.7–36.9%), and Lower (24.2–37.2%).	9.8–12.7%.
Fukuda (2016) [22]	**I**: 74.8; **C**: 75.2 (Mean)	**I**: 76 (73.1%); **C**: 52 (74.3%)	Neoplasms of the **Esophagus**,** Stomach**,** and Colon**.	Esophagus (26.8%), Stomach (46.1%), and Colon (26.9%).	100%
Kataoka (2019) [27]	**I**: 72.9; **C**: 73.0 (Mean)	NA	**Adenoma**, **Differentiated**, **Undifferentiated**, and **Mixed Carcinoma**.	Gastric **Body** (72.3%) and **Antrum** (27.7%).	35.8–40.0%
Kawata (2018) [23]	**I**: 78.5; **C**: 78 (Median)	**I**: 33 (86.8%); **C**: 37 (80.4%)	**Mucosal Carcinoma** (50%), **Adenoma** (31.8%), and **Submucosal Carcinoma** (18.2%).	Upper (18%), Middle (38%), and Lower (44%).	100%
Kikuchi (2018) [24]	Total: 75.5 ± 5.9 (Mean)	Total: 17 (85.0%)	**Mucosal Cancer** (12), **Adenoma** (7), and **Submucosal Cancer** (3).	Upper (4), Middle (10), and Lower (8).	100%
Kikuchi (2019) [25]	**I**: 75.6; **C**: 76.5 (Mean)	**I**: 31 (81.6%); **C**: 74 (87.1%)	**Intramucosal** and **Submucosal Carcinoma**.	Upper (16.3%), Middle (32.5%), and Lower (51.2%).	100%
Kim (2025) [31]	**I**: 66.0; **C**: 68.0 (Median)	**I**: 81 (64.3%); **C**: 79 (62.7%)	**Adenocarcinoma** (69.0–81.7%) and **Adenoma** (15.9–29.4%).	Lower (49.2–57.1%), Middle (33.3–34.1%), and Upper (8.8–16.7%).	16.7–23.0%
Lee (2023) [32]	**I**: 67.3; **C**: 68.1 (Mean)	**I**: 93 (74.4%); **C**: 92 (75.4%)	**Adenocarcinoma** (62.1–63.3%) and **Adenoma** (34.2–37.9%).	Lower (50.0–53.6%), Middle (41.8–42.4%), and Upper (4.0–8.2%).	35.2–39.2%
Tan (2016) [29]	Total: 55.47 ± 12.2 (Mean)	**I**: 57 (59.4%); **C**: 160 (53.2%)	**Early Gastric Cancer** and **Gastric Adenoma**.	Upper (36.8%), Middle (25.4%), and Lower (37.8%).	5.0–5.2%
Tsuji (2015) [26]	**I**: 73.6; **C**: 74.8 (Mean)	**I**: 41 (91.1%); **C**: 34 (91.9%)	**Intestinal** (94.4–100%) and **Diffuse Histology**.	Upper (19.7%), Middle (43%), and Lower (37.2%).	56.1–64.4%
Wang (2020) [30]	**I**: 60.6; **C**: 61.4 (Mean)	**I**: 85 (73.9%); **C**: 86 (74.8%)	**Differentiated** (71.3%), **Undifferentiated** (15.7–17.4%), and **Adenoma** (11.3–13.0%).	Lower (48.7–53.0%), Upper (31.3–33.9%), and Middle (15.7–17.4%).	2.6–3.5%
Zhang (2025) [20]	**I**: 64.0; **C**: 64.0 (Mean)	**I**: 71 (69.6%); **C**: 69 (67.6%)	**Adenocarcinoma** (74.8–82.9%) and **Squamous Cell Carcinoma** (3.3–14.2%).	Lower (45.0–57.3%), Middle (24.2–29.1%), Esophagus (13.7–21.9%), and Upper (4.0–4.7%).	2.9–3.9%

*I* intervention, *C* control, *M* male, *F* female, *LGD* low grade dysplasia, *HGD* high grade dysplasia, *FG* fibrin glue, *PGA* polyglycolic acid, *U* upper, *M* middle, L lower

**Table 3:** Resected specimen and tumor sizes across the included studies

Study ID	Resected Specimen Size (mm)	Tumor Size (mm)
Abiko (2021)	30.0 (Median)	13.0 (Median)
Fukuda (2016)	43.4–45.3 (Mean)	NS
Kawata (2018)	38.0–43.0 (Median)	NS
Kikuchi (2018)	31.5 (Mean)	14.0 (Mean)
Kikuchi (2019)	NS	12.6–16.6 (Mean)
Tsuji (2015)	40.1–43.9 (Mean)	NS
Kataoka (2019)	47.5–51.3 (Mean)	23.8–26.9 (Mean)
Chen (2025)	52.0–53.0 (Median)	NS (Inclusion 30 mm)
Tan (2016)	26.9–43.8% are 40 mm	NS
Wang (2020)	44.0–46.6 (Mean)	NS
Kim (2025)	43.0–44.0 (Median)	14.0–15.0 (Median)
Zhang (2025)	NS	3.2–4.1 (Median)
Lee (2023)	44.3–44.5 (Mean)	20.2–22.5 (Mean)

### Risk of Bias Assessment

**Fig. 2:**
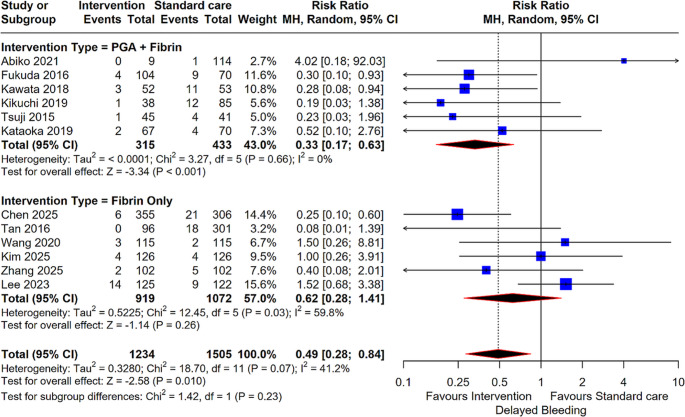
Forest plot for Delayed Bleeding. Comparison of intervention (PGA + Fibrin and Fibrin Only) versus standard care for delayed bleeding risk

The risk of bias of the included RCTs was assessed using the Cochrane RoB 2 tool. All trials were judged as having some concerns overall, mainly due to concerns in deviations from intended interventions (D2), while the remaining domains were largely rated as low risk (Fig. 2). For the observational studies assessed using the Newcastle–Ottawa Scale (NOS), four studies (Chen 2025, Kikuchi 2019, Wang 2020, and Zhang 2025) were rated as good quality (9 points), whereas five studies (Fukuda 2016, Kawata 2018, Tan 2016, Tsuji 2015, and Abiko 2021) were rated as poor quality (6–7 points), primarily due to limited comparability Table 4.

**Table 4:** Quality assessment of observational studies using NOS

Study ID	Selection	Comparability	Outcome	Overall	Final decision
Chen 2025	4	2	3	9	Good quality
Fukuda 2016	4	0	2	6	Poor quality
Kawata 2018	4	0	2	6	Poor quality
Kikuchi 2019	4	2	3	9	Good quality
Tan 2016	4	0	3	7	Poor quality
Wang 2020	4	2	3	9	Good quality
Zhang 2025	4	2	3	9	Good quality
Tsuji 2015	4	0	2	6	Poor quality
Abiko 2021	4	0	3	7	Poor quality

### Bleeding Outcomes

#### Delayed Bleeding

**Fig. 3:**
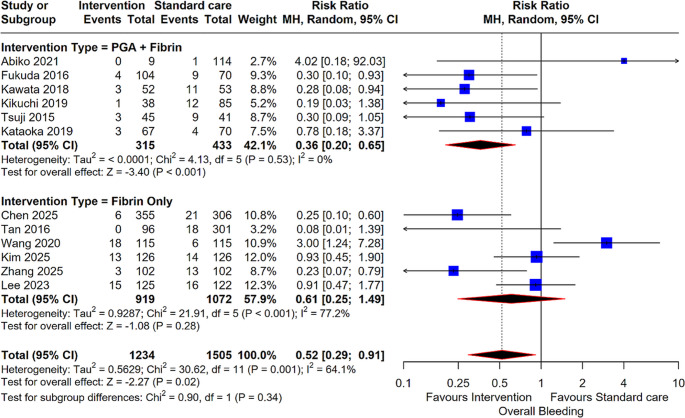
Forest plot for Overall Bleeding. Comparison of intervention (PGA + Fibrin and Fibrin Only) versus standard care for overall bleeding risk

In an analysis of 12 studies involving 2,739 patients, the meta-analysis indicated that the intervention significantly reduced the risk of delayed bleeding compared to standard care (RR: 0.49; 95% CI: 0.28, 0.84; *P* = 0.01). This reduction was particularly robust in the PGA + Fibrin subgroup (6 studies), which yielded a highly significant result (RR: 0.33; 95% CI: 0.17, 0.63; *P* < 0.001). In contrast, the Fibrin Only subgroup (6 studies) did not demonstrate a statistically significant reduction in bleeding risk (RR: 0.62; 95% CI: 0.28, 1.41; *P* = 0.26) (Fig. 3).

#### Overall Bleeding

**Fig. 4:**
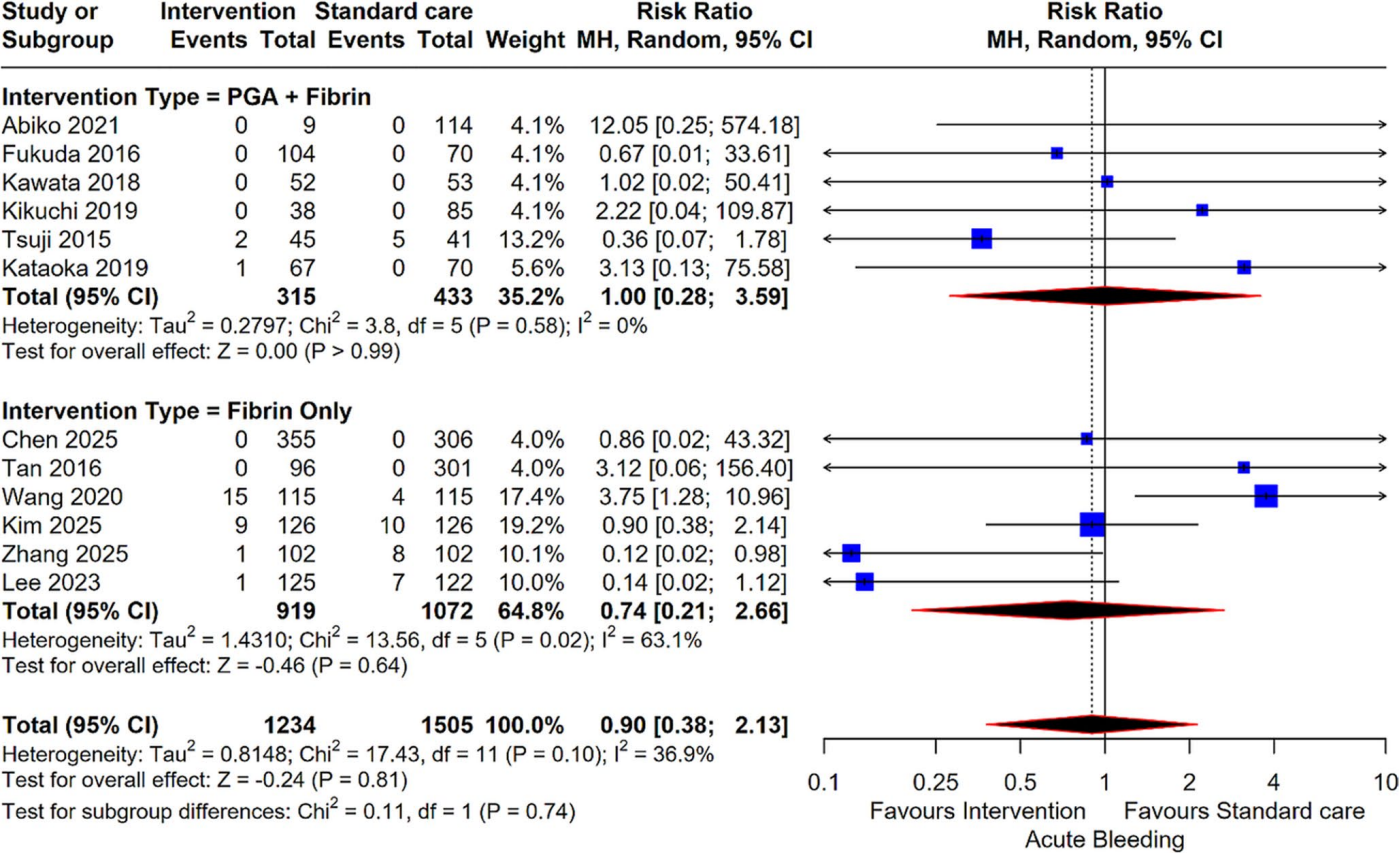
Forest plot for Acute Bleeding. Comparison of intervention (PGA + Fibrin and Fibrin Only) versus standard care for acute bleeding risk

Across 12 studies (2,739 patients), there was a significant overall reduction in the risk of overall bleeding for the intervention group (RR: 0.52; 95% CI: 0.29, 0.91; *P* = 0.02). The PGA + Fibrin subgroup (6 studies) again showed a significant benefit (RR: 0.36; 95% CI: 0.20, 0.65; *P* < 0.001), while the Fibrin Only subgroup (6 studies) showed no statistically significant difference compared to standard care (RR: 0.61; 95% CI: 0.25, 1.49; *P* = 0.28) (Fig. 4).

#### Acute Bleeding

**Fig. 5:**
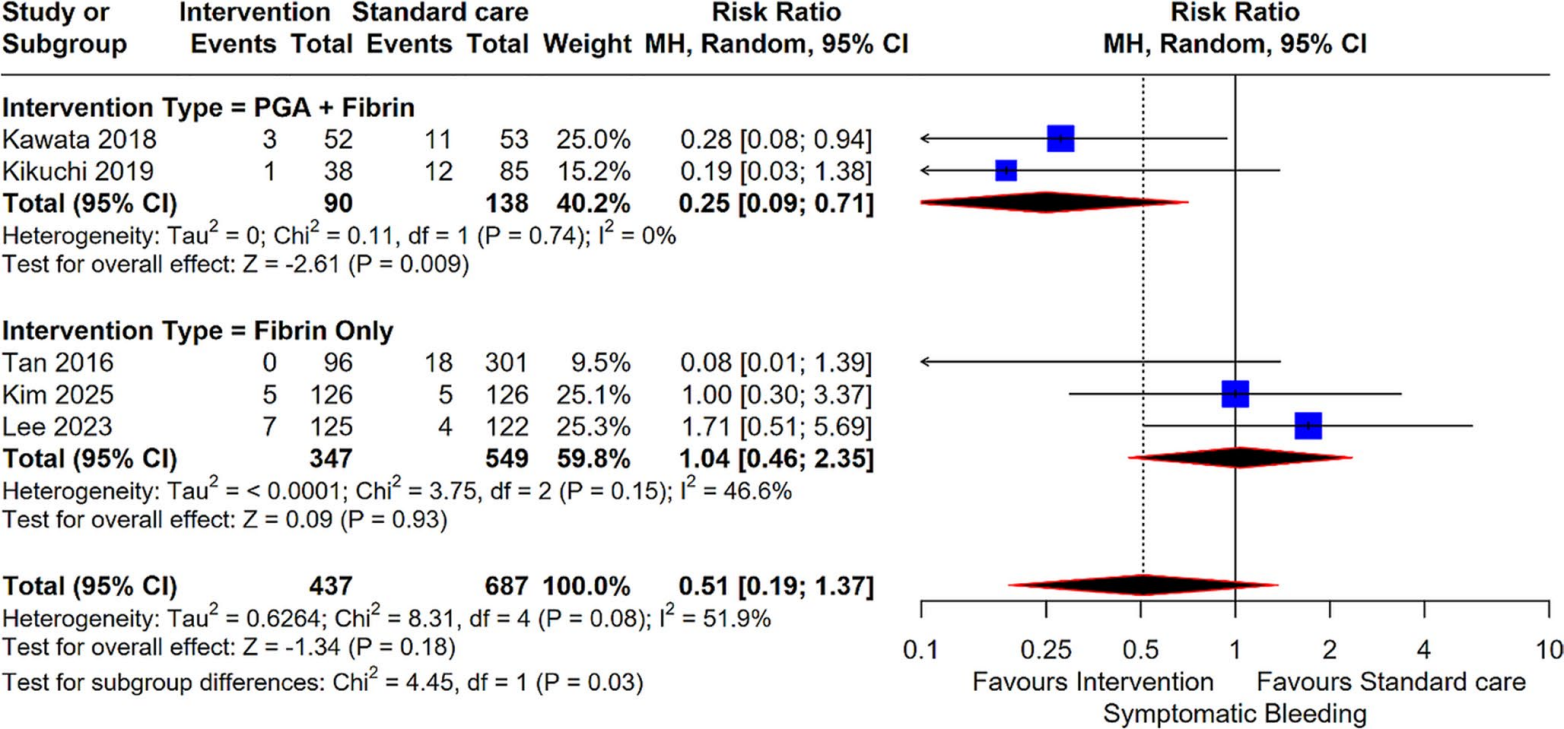
Forest plot for Symptomatic Bleeding. Comparison of intervention (PGA + Fibrin and Fibrin Only) versus standard care for symptomatic bleeding risk

For the outcome of acute bleeding, no statistically significant difference was found between the intervention and standard care groups overall in an analysis of 12 studies (2,739 patients) (RR: 0.90; 95% CI: 0.38, 2.13; *P* = 0.81). Neither the PGA + Fibrin subgroup (6 studies; RR: 1.00; *P* > 0.99) nor the Fibrin Only subgroup (6 studies; RR: 0.74; *P* = 0.64) demonstrated a significant reduction in acute bleeding events (Fig. 5).

#### Symptomatic Bleeding

**Fig. 6:**
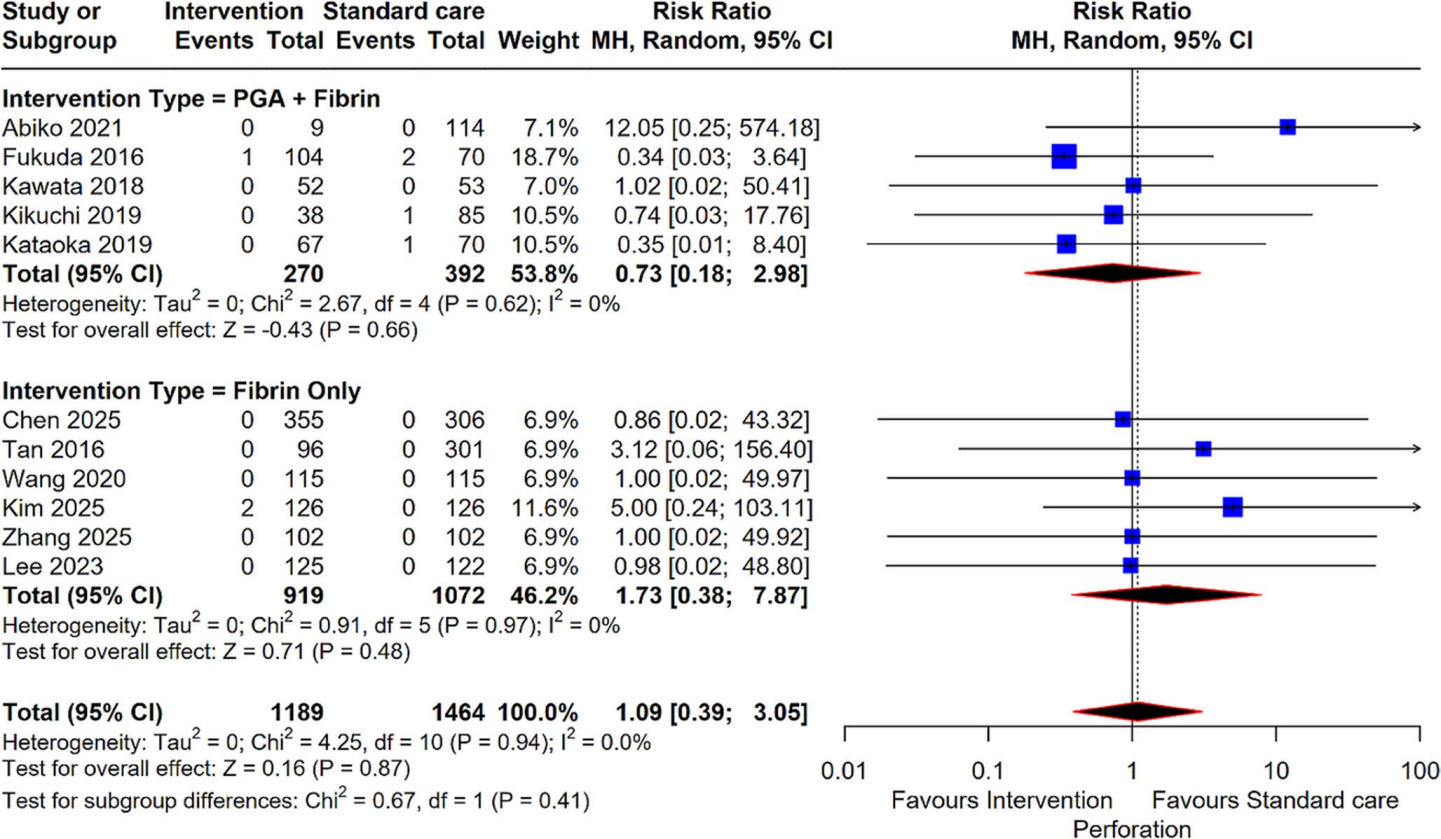
Forest plot for Perforation. Comparison of intervention (PGA + Fibrin and Fibrin Only) versus standard care for the risk of procedural perforation

The analysis for symptomatic bleeding, encompassing 5 studies and 1,124 patients, did not show a statistically significant difference between the groups overall (RR: 0.51; 95% CI: 0.19, 1.37; *P* = 0.18). However, the PGA + Fibrin subgroup (2 studies) exhibited a significant reduction in symptomatic bleeding events (RR: 0.25; 95% CI: 0.09, 0.71; *P* = 0.009). The Fibrin Only subgroup (3 studies) showed no benefit (RR: 1.04; *P* = 0.93), resulting in a significant difference between these two subgroups (*P* = 0.03) (Fig. 6).

### Complication Outcomes

#### Perforation

**Fig. 7:**
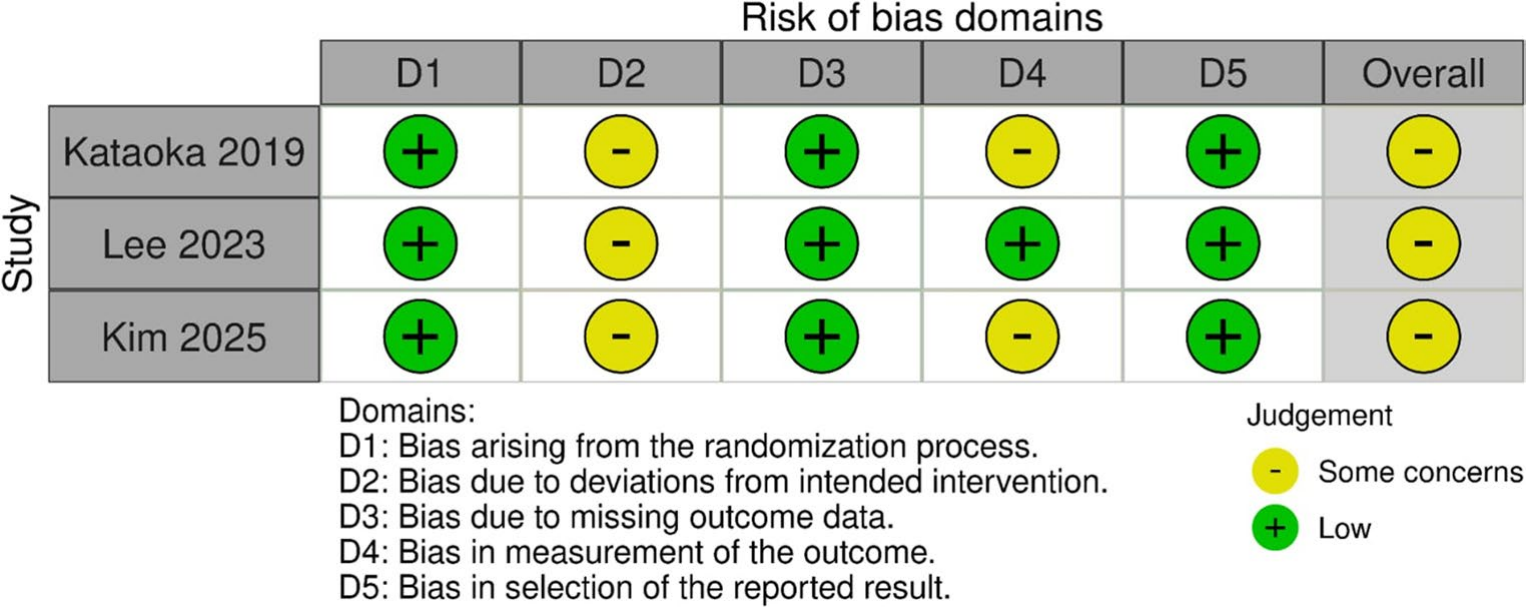
Quality assessment of randomized controlled trials using ROB-2 tool

Regarding procedural complications, an analysis of 11 studies involving 2,653 patients found no significant difference in the risk of perforation between the intervention and standard care groups (RR: 1.09; 95% CI: 0.39, 3.05; *P* = 0.87). Heterogeneity for this outcome was very low (I^2^ = 0%), and neither the PGA + Fibrin subgroup (5 studies; RR: 0.73; *P* = 0.66) nor the Fibrin Only subgroup (6 studies; RR: 1.73; *P* = 0.48) showed a significant change in perforation risk (Fig. 7).

## Discussion

This systematic review and meta-analysis assessed the effectiveness of fibrin glue, with or without polyglycolic acid (PGA) sheets, in preventing post-ESD bleeding for gastric tumors. Across thirteen studies with 2,728 patients, fibrin-based therapy significantly reduced overall and delayed bleeding compared with standard care. The benefit was primarily driven by the combination of PGA sheets and fibrin glue, which consistently outperformed fibrin alone across bleeding outcomes, including symptomatic events. In contrast, fibrin alone did not demonstrate a consistent protective effect. No significant reduction was observed in acute bleeding, and perforation rates were similar between groups. Although effect estimates favored the intervention at both 4- and 8-week follow-up, no significant interaction by duration was detected, indicating a stable protective effect during the early post-ESD healing phase.

The biological rationale for using PGA sheets and fibrin glue after gastric ESD is grounded in the concept of tissue shielding and enhanced hemostatic stabilization. ESD creates a large artificial ulcer with exposed submucosal vessels that remain vulnerable to acid, bile reflux, and mechanical irritation, predisposing to delayed bleeding. PGA sheets act as a bioabsorbable mechanical barrier that physically covers the ulcer bed, protecting exposed vessels from chemical and mechanical insults while promoting granulation tissue formation and re-epithelialization [27]. Fibrin glue, composed of fibrinogen and thrombin, mimics the final step of the coagulation cascade, forming a stable fibrin clot that adheres to tissue surfaces, seals microvessels, and reinforces hemostasis at the ulcer base [29, 31]. However, fibrin alone may be susceptible to early degradation in the gastric environment. The combined application of PGA sheets with fibrin glue provides synergistic benefit; the sheet offers structural support and sustained coverage, while fibrin acts as both an adhesive and a biologic sealant that secures the patch to the ulcer surface. This dual mechanism: mechanical shielding plus biologically enhanced clot stabilization, likely explains the superior reduction in delayed bleeding observed with combination therapy compared with fibrin alone.

Post-endoscopic submucosal dissection bleeding is commonly classified by timing and clinical presentation into acute, delayed, and symptomatic events, which reflect distinct pathophysiological processes and clinical implications [33, 34]. Acute bleeding typically occurs within the first 24–48 h after ESD and is often related to intra-procedural vessel injury or insufficient immediate hemostasis, whereas delayed bleeding arises days to weeks post-procedure and is influenced by ongoing ulcer exposure to gastric acid, mechanical stress, and the resumption of antithrombotic therapy as coagulum stabilizes and systemic hemostatic balance shifts post-discharge [35]. Delayed bleeding events may also be associated with ulcer sloughing and the breakdown of early clot formation at the resection site [34, 36].

These mechanisms partly explain why Tissue-shielding strategies, particularly PGA sheets combined with fibrin glue, are more effective in preventing delayed rather than acute bleeding after ESD. By providing sustained ulcer coverage during the vulnerable healing phase, they stabilize hemostasis, protect against acid and mechanical stress, and reduce bleeding risk, consistent with our meta-analysis findings.

Compared with the earlier meta-analysis by Li et al. [37], which included four studies (212 PGA-treated vs. 208 controls) and demonstrated a significant reduction in overall post-ESD bleeding (RR 0.33, 95% CI: 0.16–0.69), our study expands and refines the available evidence. While Li et al. focused exclusively on PGA sheets in high-risk early gastric cancer populations, particularly patients receiving antithrombotic therapy, our meta-analysis incorporates a substantially larger and more contemporary dataset, including recent multicenter RCTs and propensity-matched studies. Consistent with their findings, we observed a significant reduction in overall and delayed bleeding, particularly with PGA plus fibrin combinations. However, our analysis further distinguishes between acute, delayed, and symptomatic bleeding and demonstrates that the protective effect is primarily driven by delayed bleeding reduction. Moreover, unlike the earlier study, we show that fibrin alone does not consistently confer benefit, thereby clarifying that the mechanical shielding component appears central to efficacy. These findings strengthen and extend prior conclusions by providing broader generalizability and more granular outcome assessment.

### Limitations and Future Research

This meta-analysis has several limitations that should be considered when interpreting the findings. First, although randomized controlled trials were included, the majority of studies were retrospective and single-center in design, which increases susceptibility to selection bias, confounding, and variability in clinical practice. Second, heterogeneity was moderate in several bleeding outcomes, likely reflecting differences in patient populations, lesion characteristics, antithrombotic management, operator expertise, and definitions of bleeding events. Third, outcomes were variably reported per patient, lesion, or ulcer, potentially introducing unit-of-analysis inconsistencies also the relatively small number of included studies limited the ability to perform meta-regression analyses to assess the potential impact of baseline covariates on outcomes. Follow-up durations were relatively short (mostly four to eight weeks), limiting assessment of longer-term complications or rebleeding risk. In addition, subgroup analyses particularly for symptomatic bleeding were based on a limited number of studies and were occasionally sensitive to single-study effects. Cost considerations and procedural time were also inconsistently reported, precluding formal evaluation of cost-effectiveness.

Furthermore, substantial procedural heterogeneity existed across studies. Variations in PGA sheet delivery (e.g., forceps vs. clip-and-pull), supplementary clipping, and fibrin glue application (source, volume, and technique) may influence mucosal shield quality, representing a limitation of our pooled analysis.

Future research should prioritize large, well-designed multicenter randomized trials with standardized bleeding definitions and uniform follow-up protocols. Comparative effectiveness research evaluating PGA plus fibrin versus other preventive strategies, alongside cost-effectiveness analyses, will further clarify the optimal approach for routine clinical practice.

## Conclusion

This meta-analysis of 13 studies (2,728 patients) concludes that combining polyglycolic acid (PGA) sheets with fibrin glue significantly reduces delayed bleeding after gastric ESD (RR 0.33), whereas fibrin glue alone is not effective. The intervention does not impact acute bleeding or perforation rates. This “tissue-shielding” approach is most beneficial for high-risk patients, such as those on antithrombotic therapy or with large lesions.

## Supplementary Information

Below is the link to the electronic supplementary material.

Supplementary Material 1

## Data Availability

No datasets were generated or analysed during the current study.
