# Supplementary material for: Efficacy of Fibrin Sealants and Polyglycolic Acid Sheets in Reducing Postoperative Hemorrhage Following Gastric Endoscopic Submucosal Dissection: A Meta-analysis of Randomized and Observational Studies
Source: J Gastrointest Cancer. 2026 Jun 5;57(1):136. doi: 10.1007/s12029-026-01502-1 (PMC13241412; doi:10.1007/s12029-026-01502-1)

**Supplementary Figures**

**Figure S1. Overall bleeding - subgroup analysis by follow-up duration.**


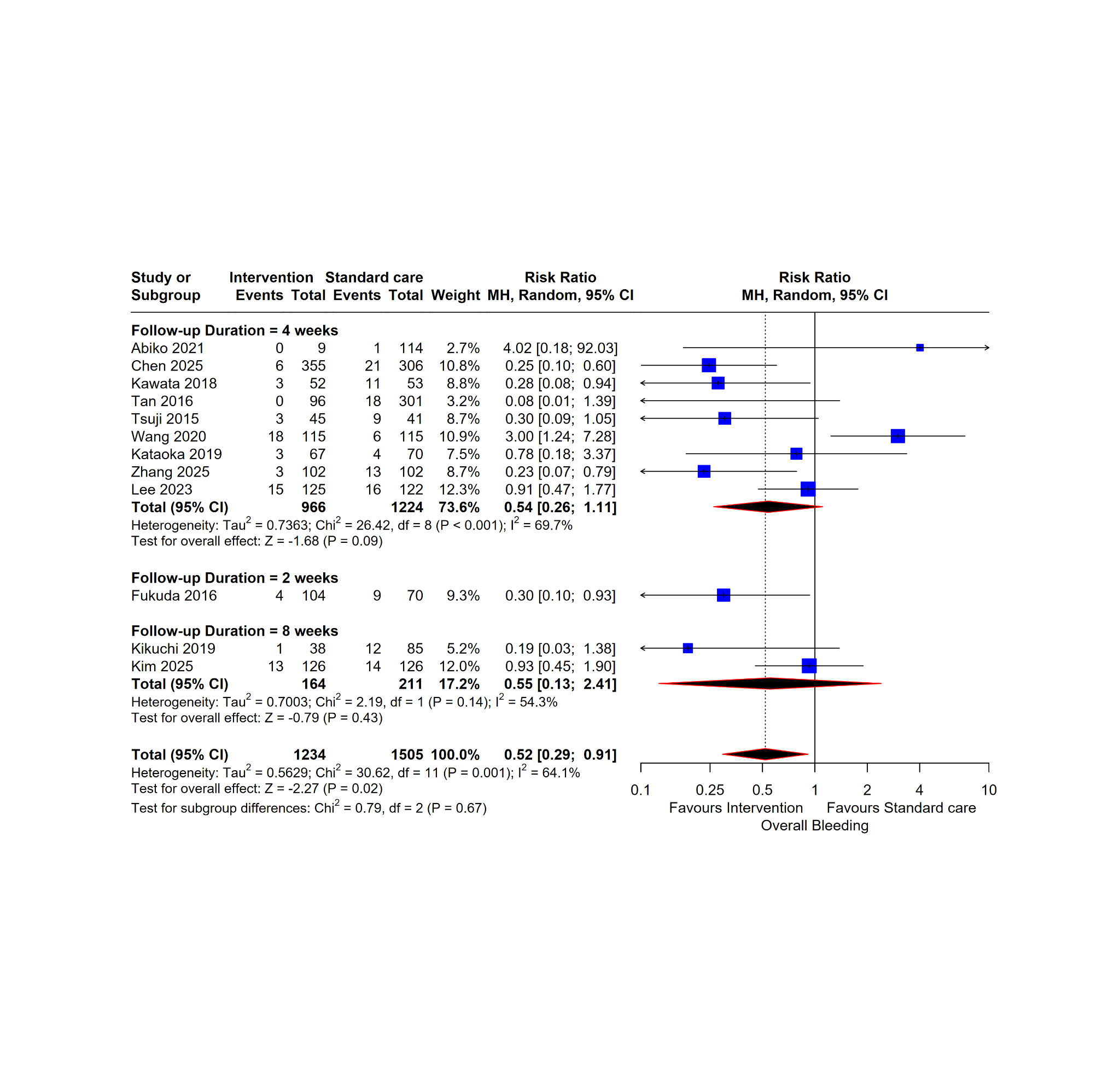


**Figure S2. Delayed bleeding - subgroup analysis by follow-up duration.**


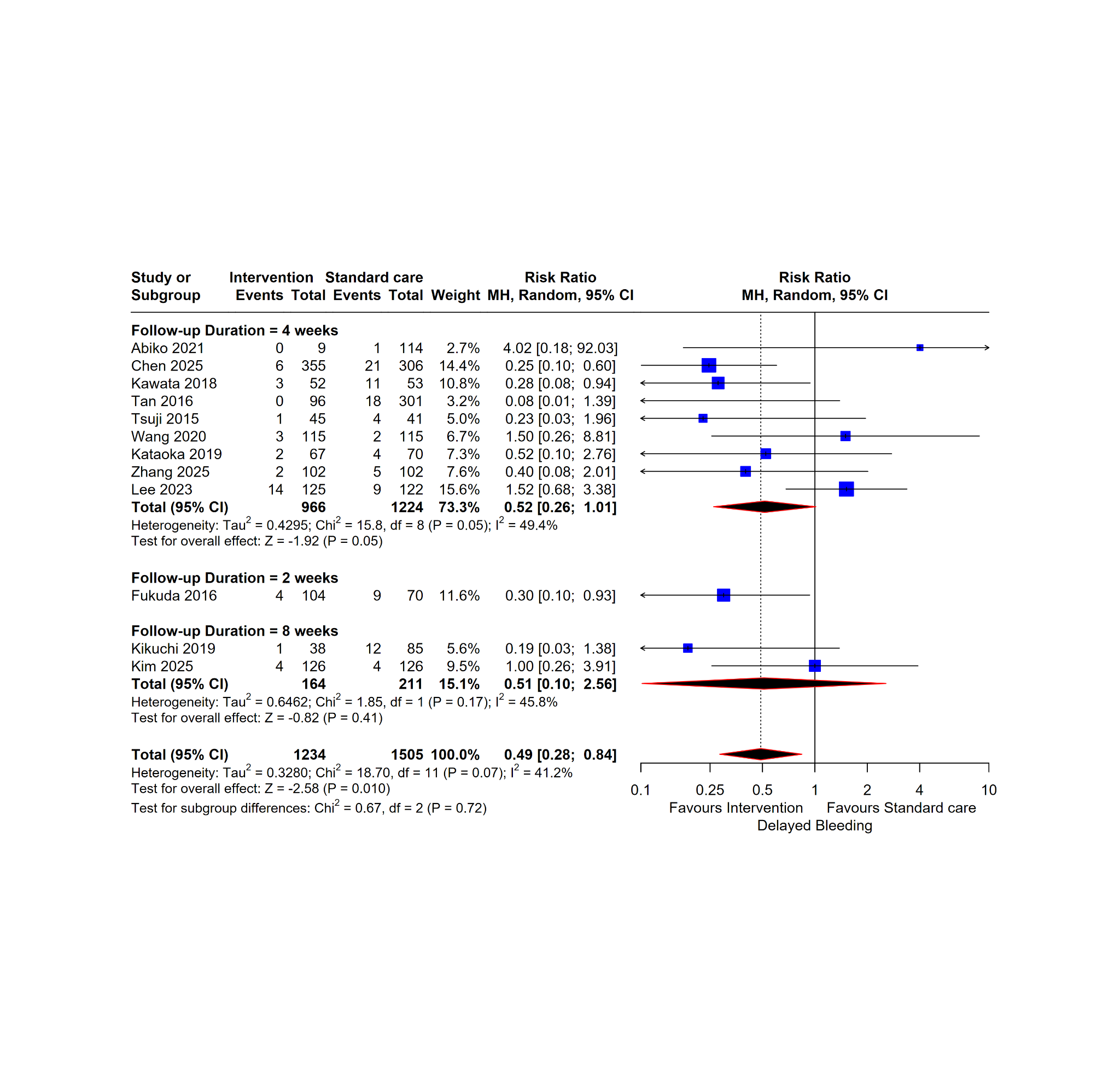


**Figure S3. Acute bleeding - subgroup analysis by follow-up duration.**


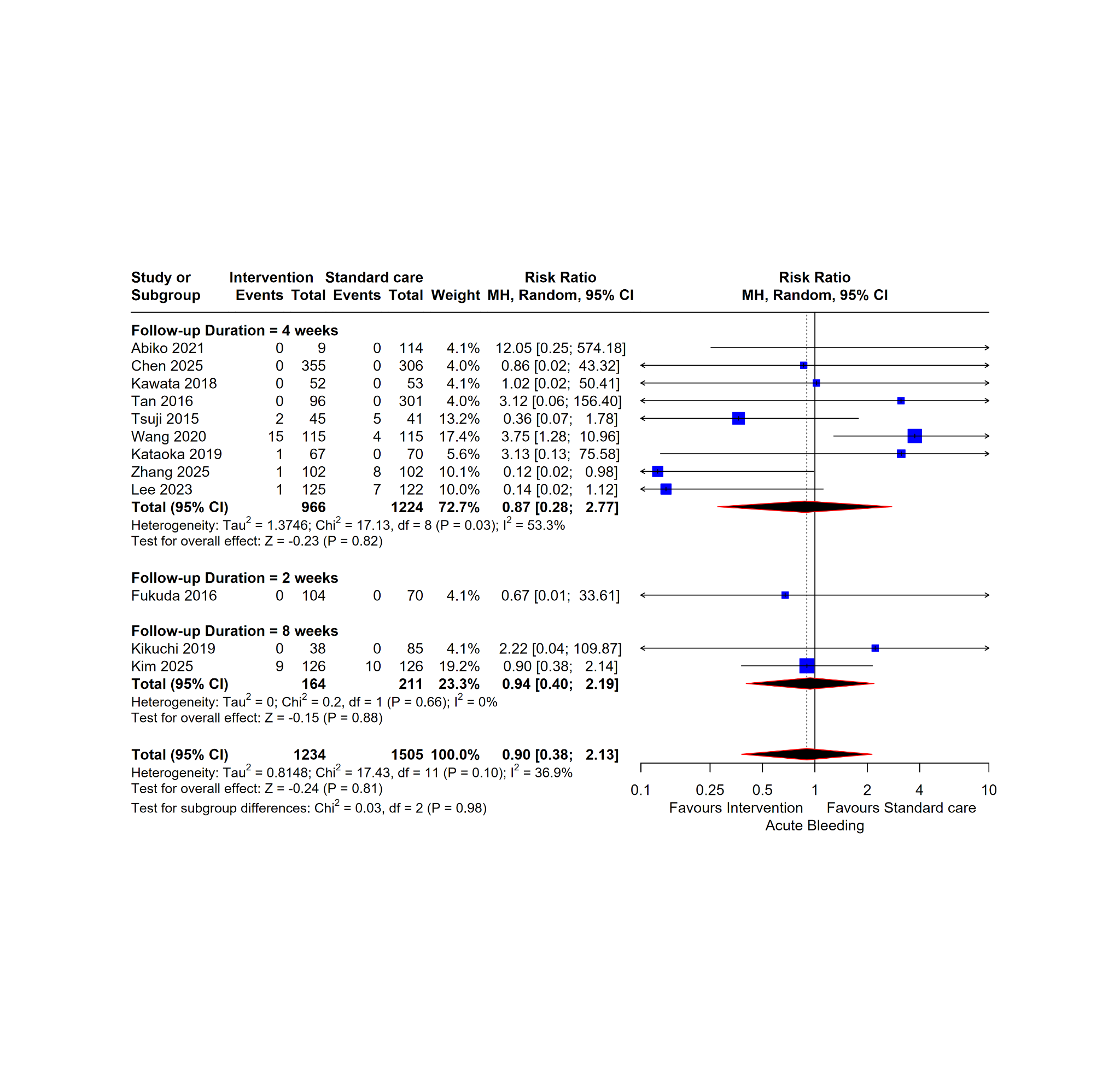


**Figure S4. Symptomatic bleeding - subgroup analysis by follow-up duration.**


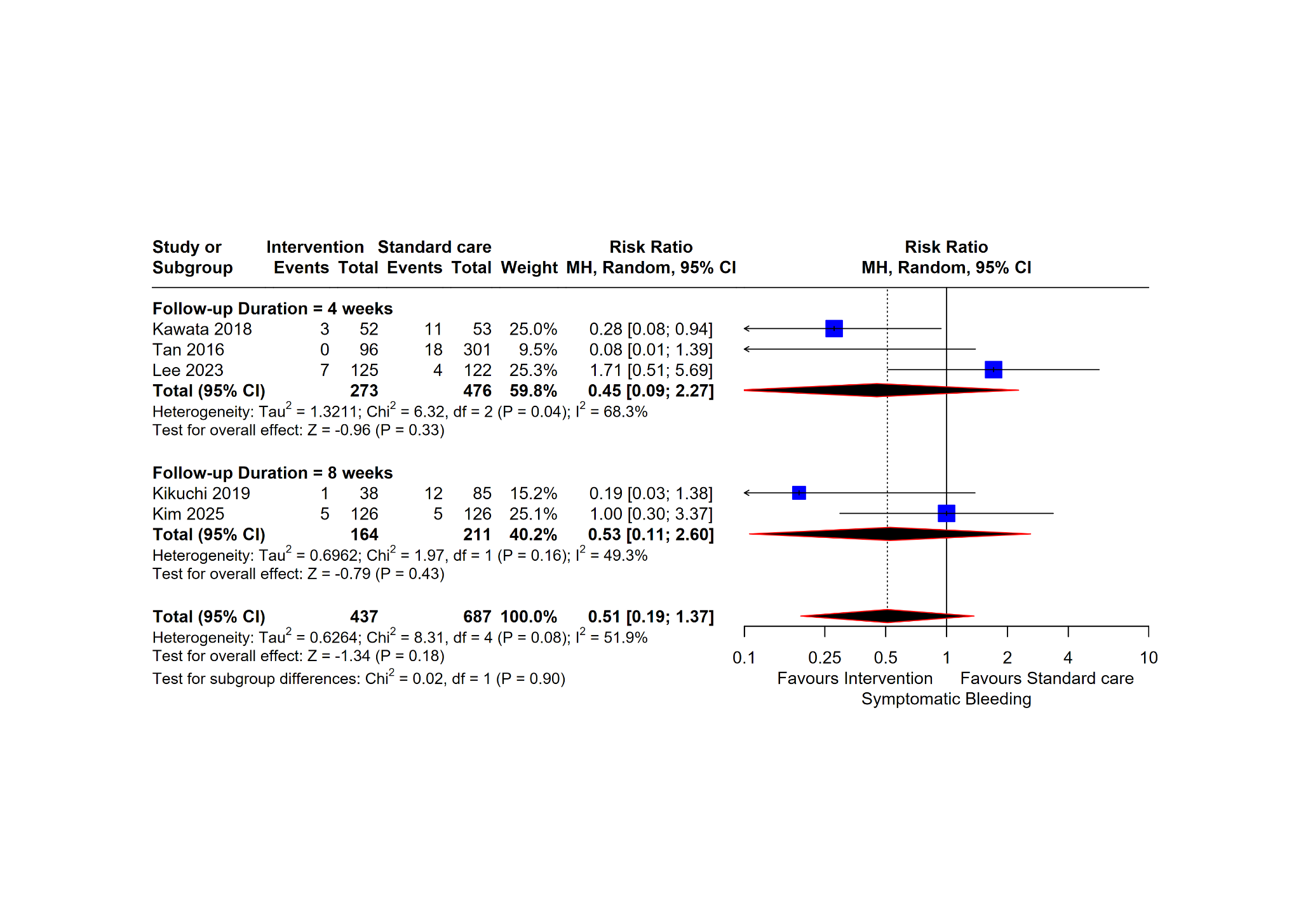


**Figure S5. Perforation - subgroup analysis by follow-up duration.**


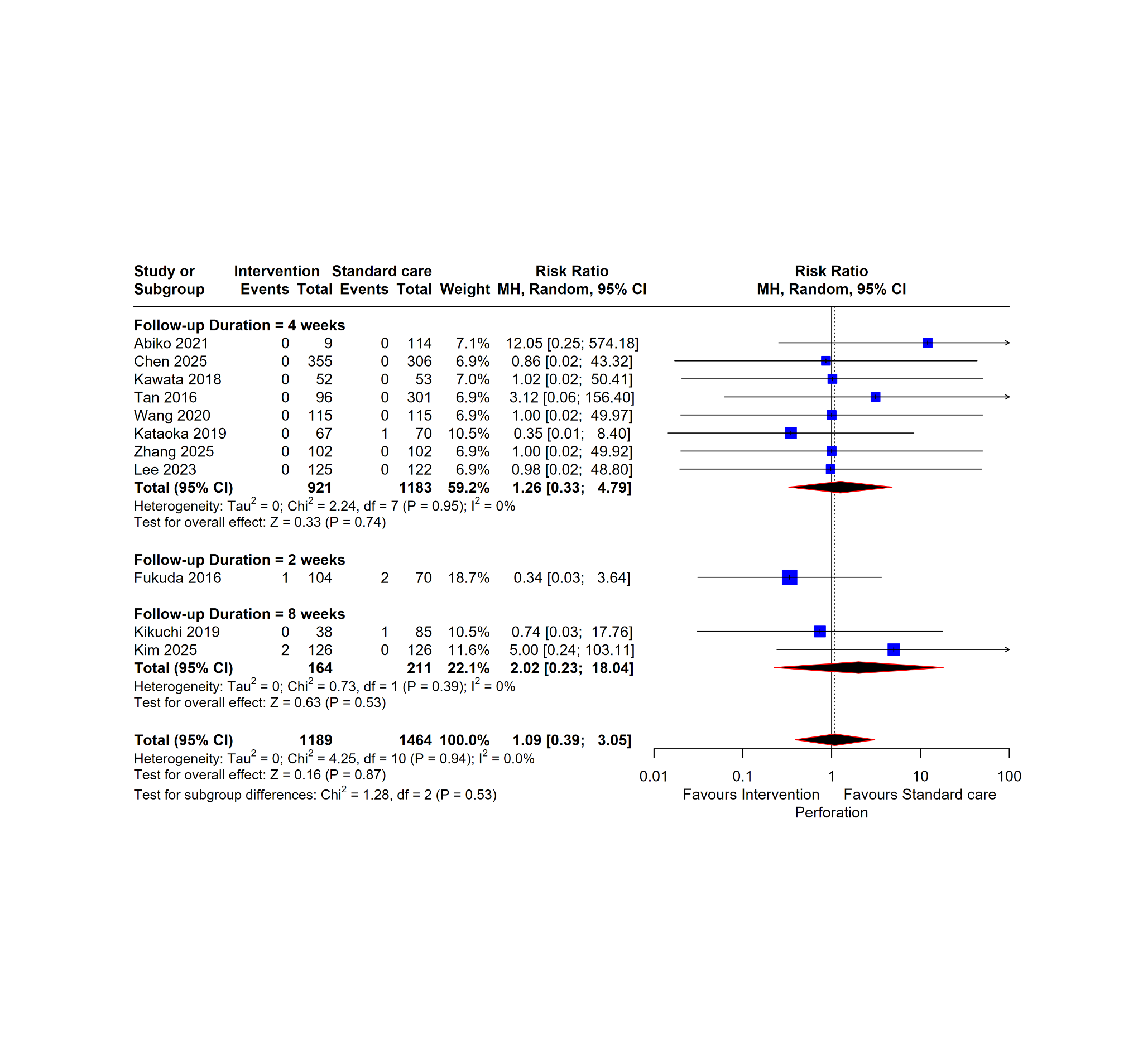


**Figure S6. Overall bleeding - leave-one-out sensitivity analysis.**


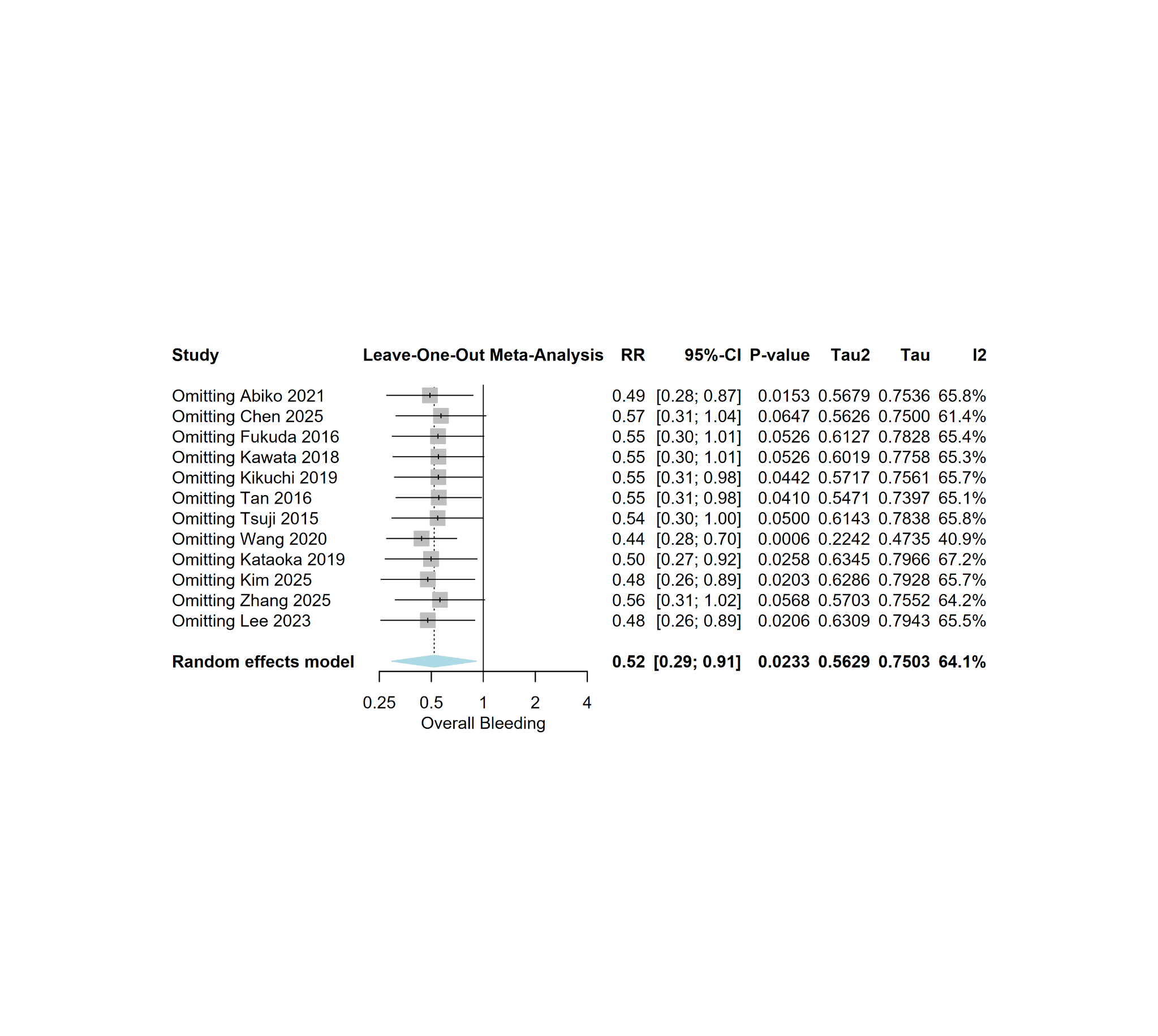


**Figure S7. Delayed bleeding - leave-one-out sensitivity analysis.**


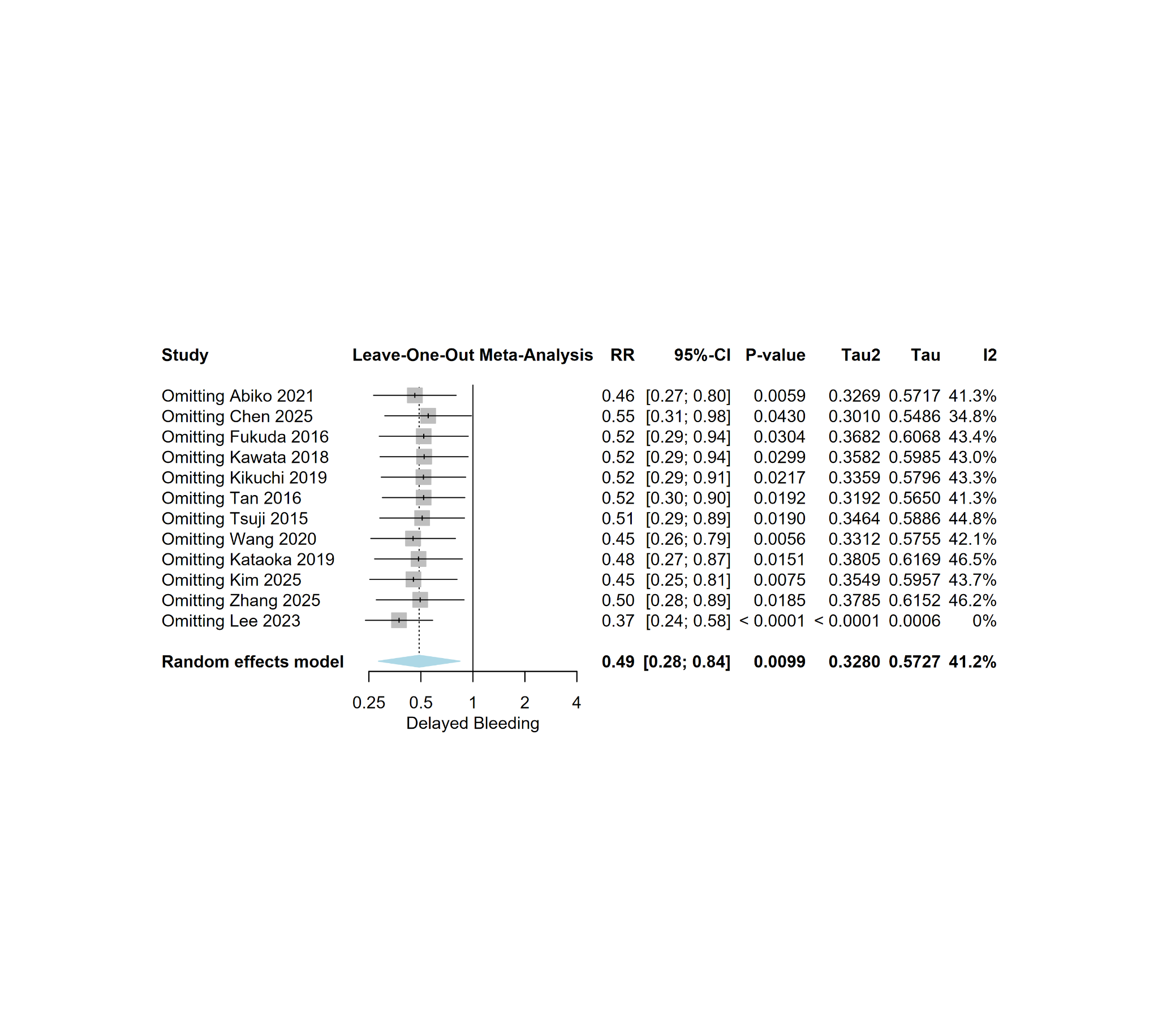


**Figure S8. Acute bleeding - leave-one-out sensitivity analysis.**


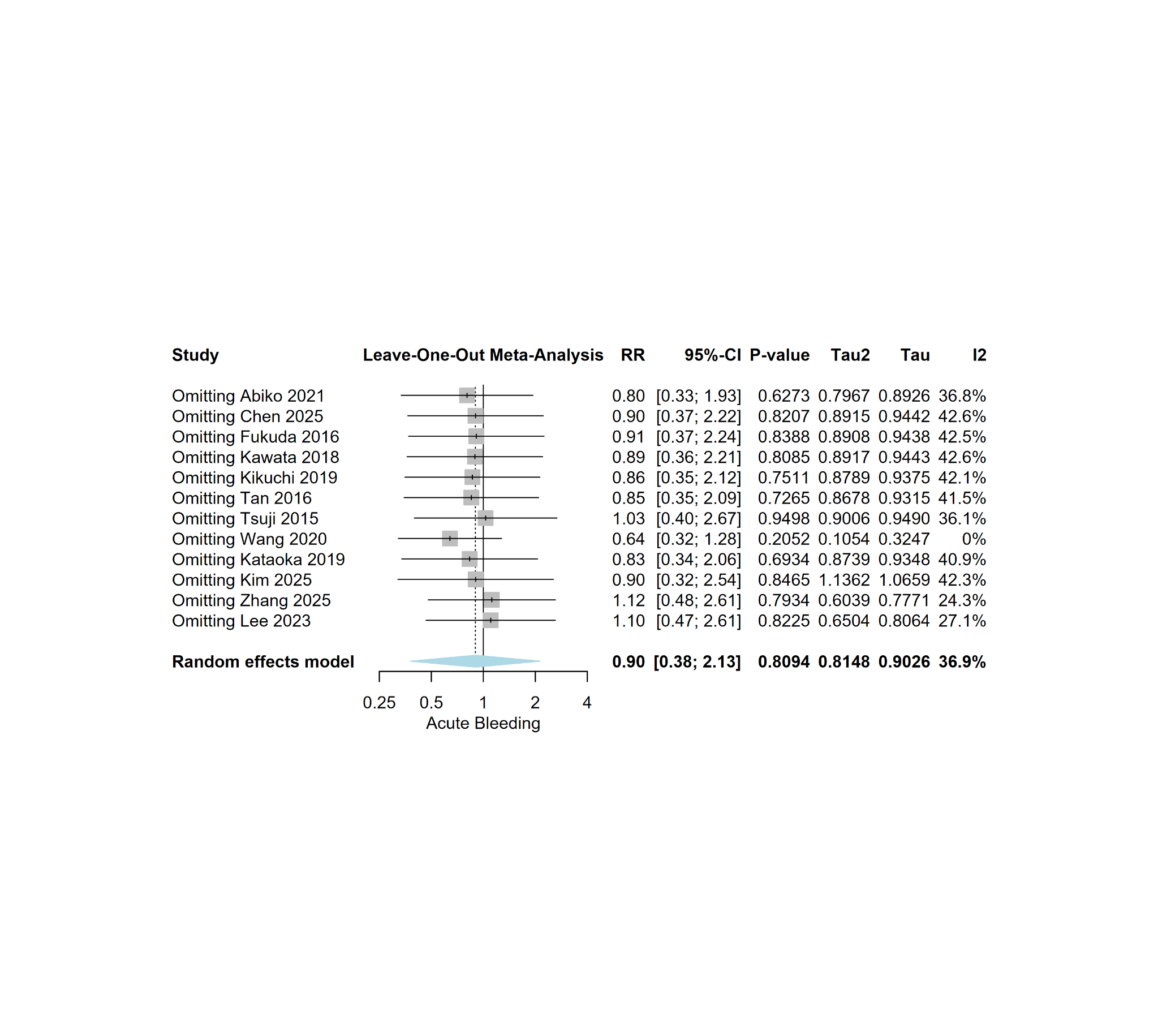


**Figure S9. Symptomatic bleeding - leave-one-out sensitivity analysis.**


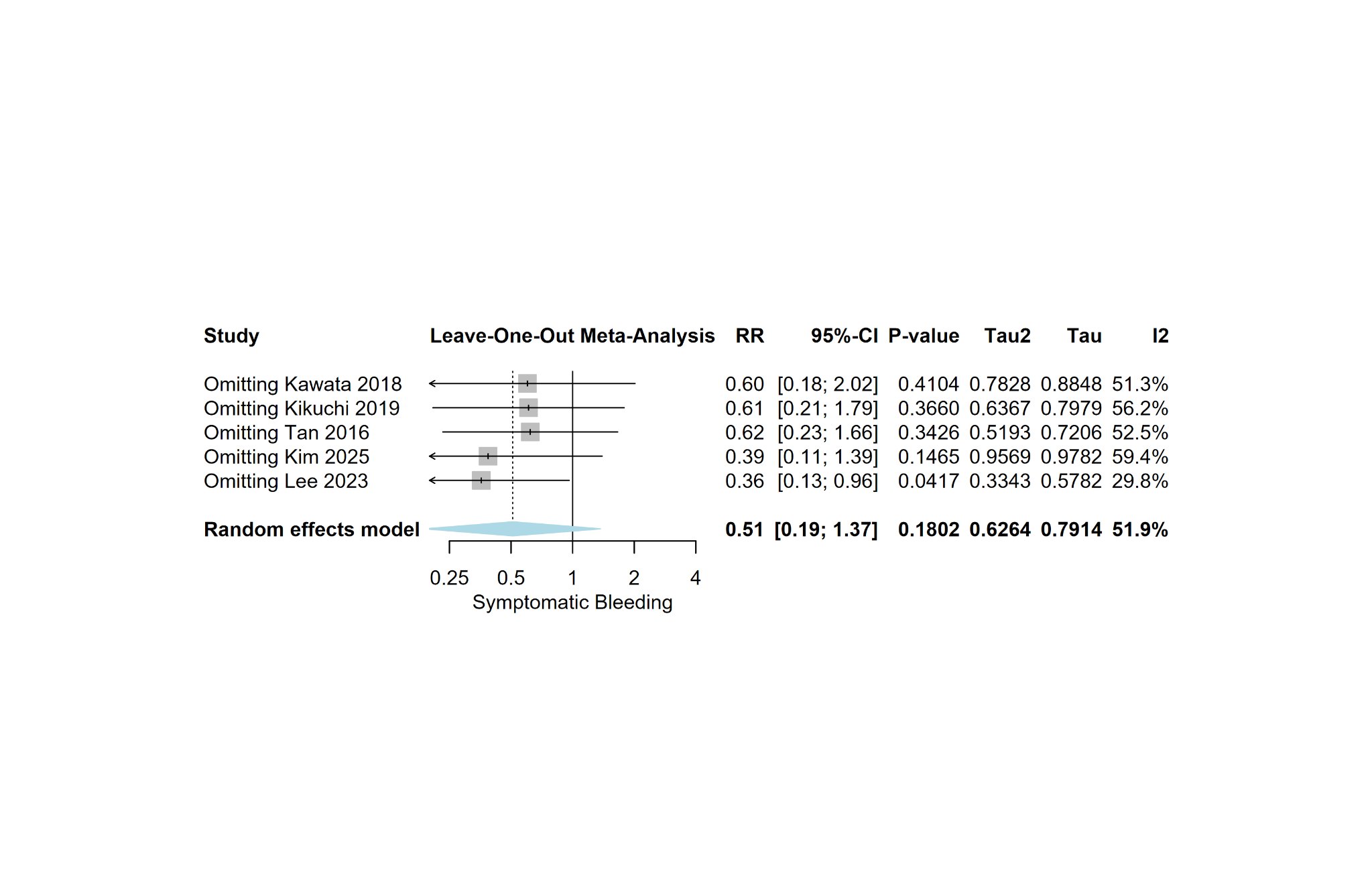


**Figure S10. Perforation - leave-one-out sensitivity analysis.**


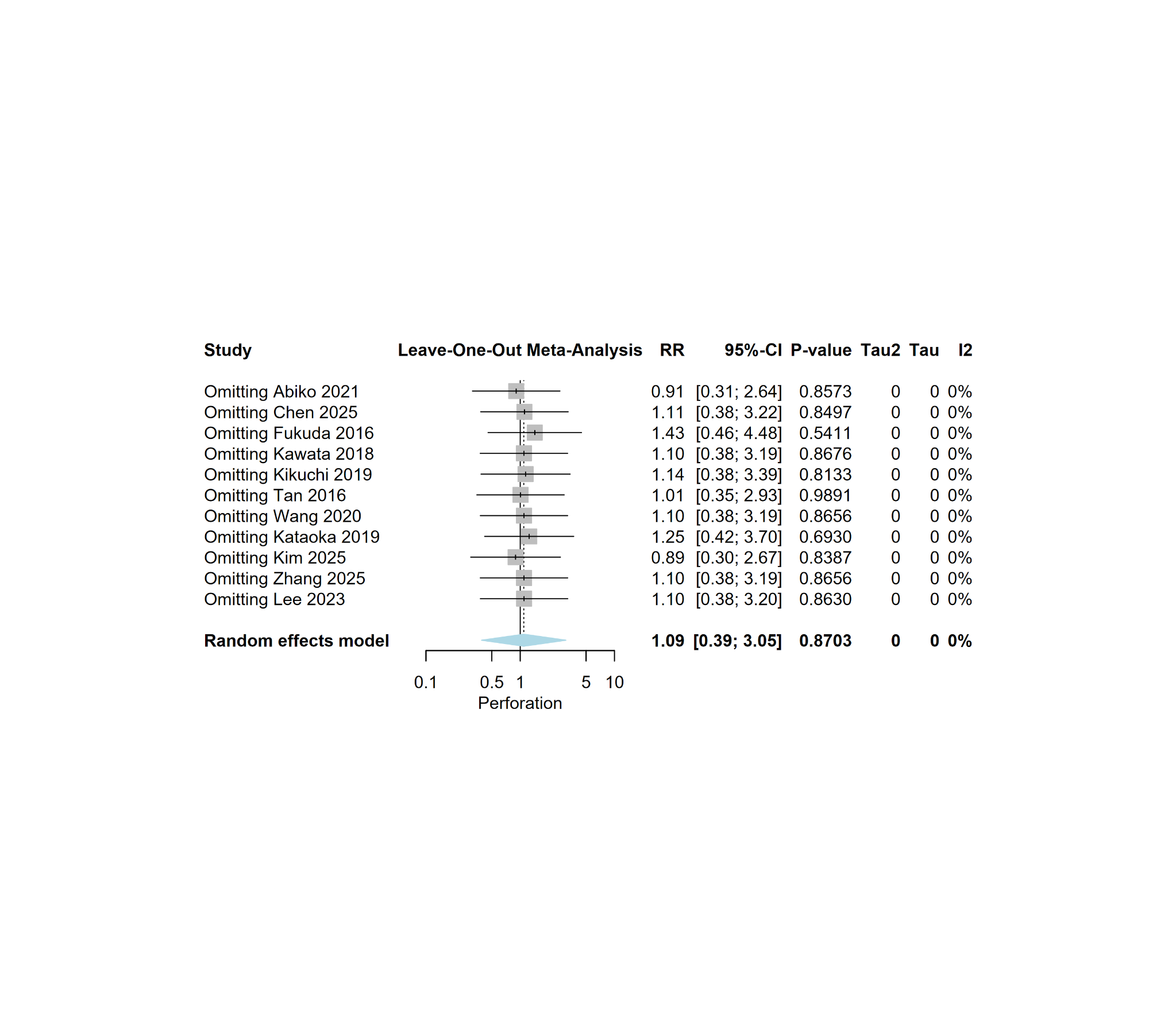


**Figure S11. Overall bleeding - funnel plot (continuity correction = 0.5).**


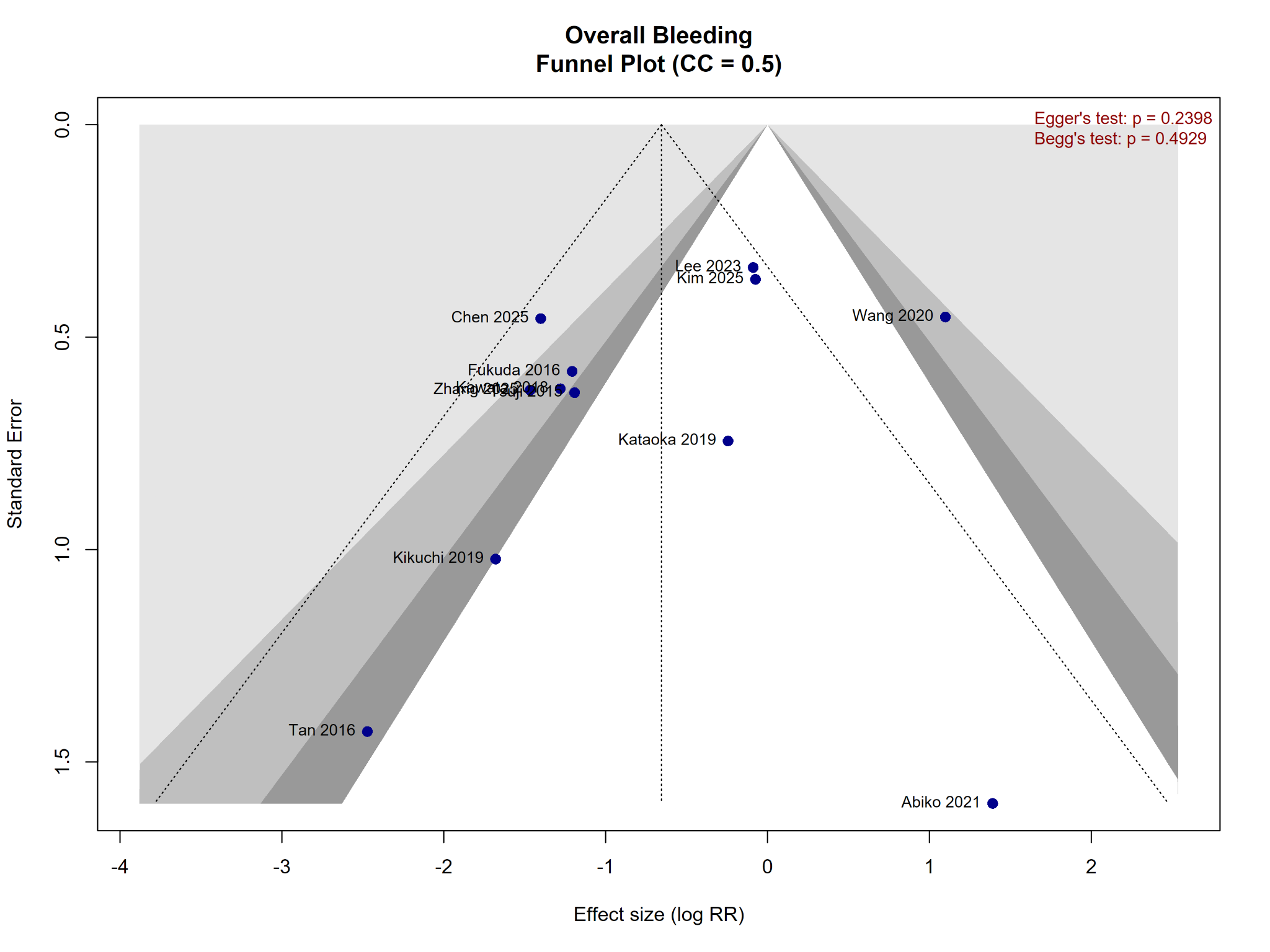


**Figure S12. Delayed bleeding - funnel plot (continuity correction = 0.5).**


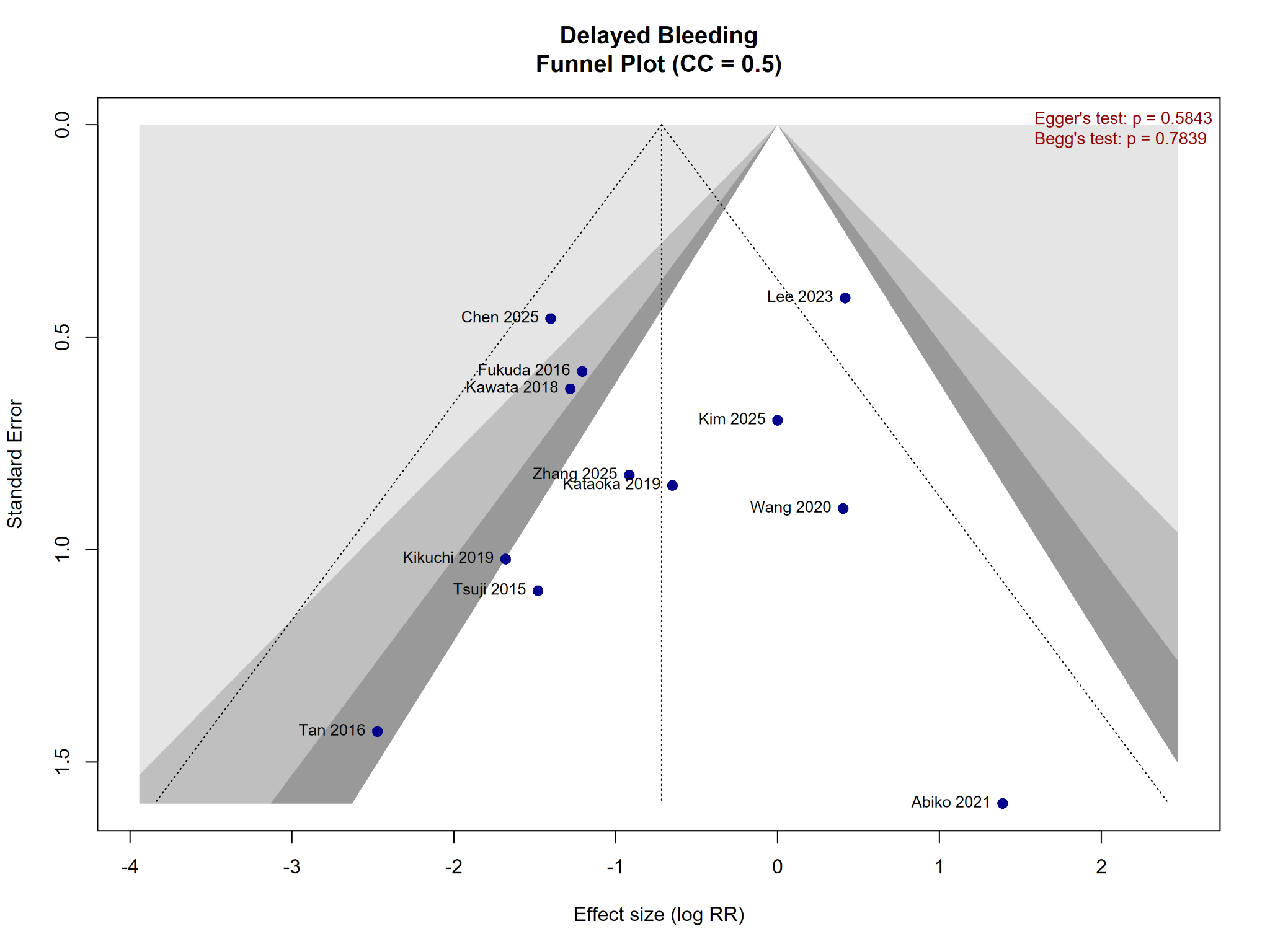


**Figure S13. Acute bleeding - funnel plot (continuity correction = 0.5).**


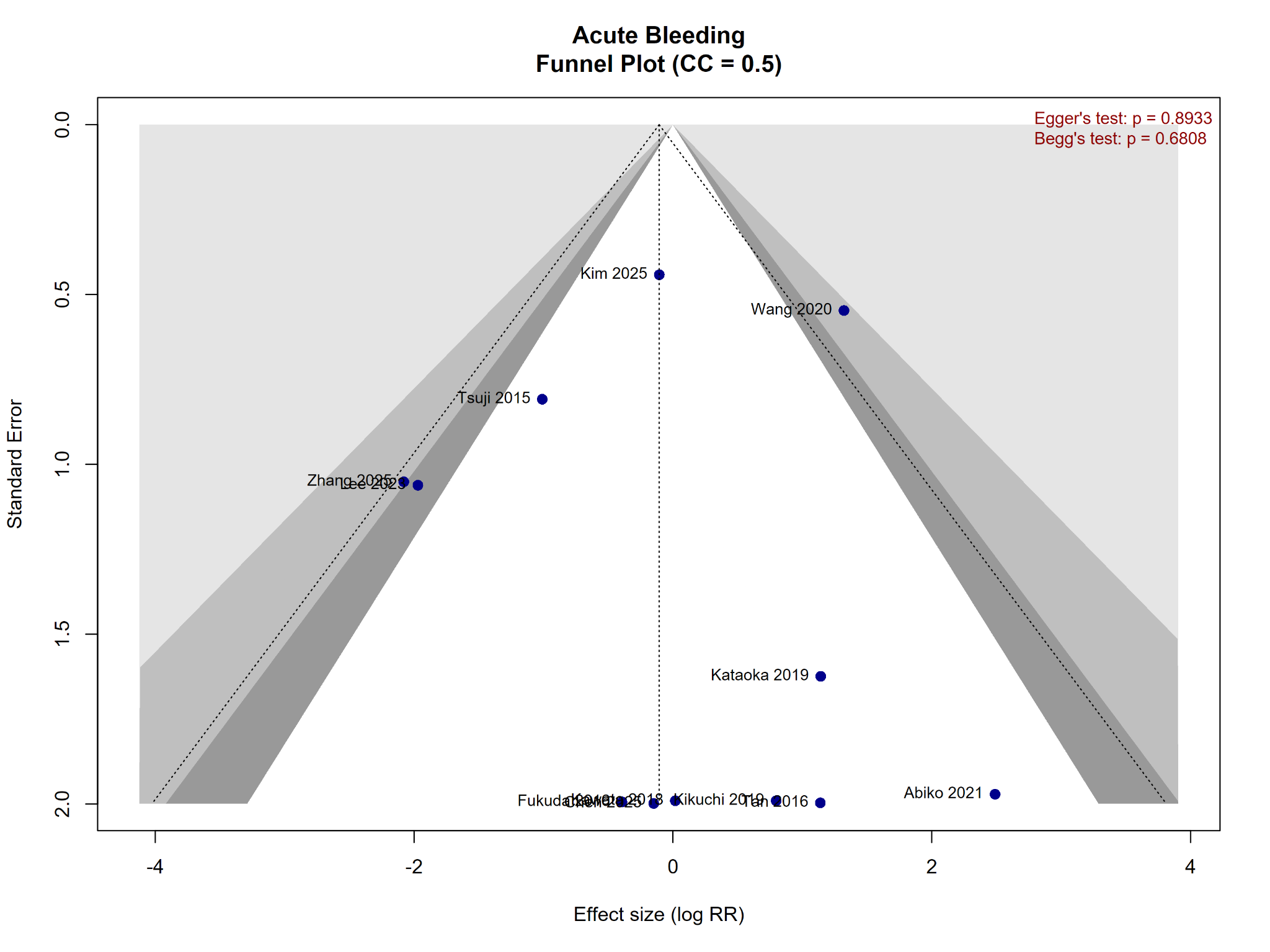


**Figure S14. Symptomatic bleeding - funnel plot (continuity correction = 0.5).**


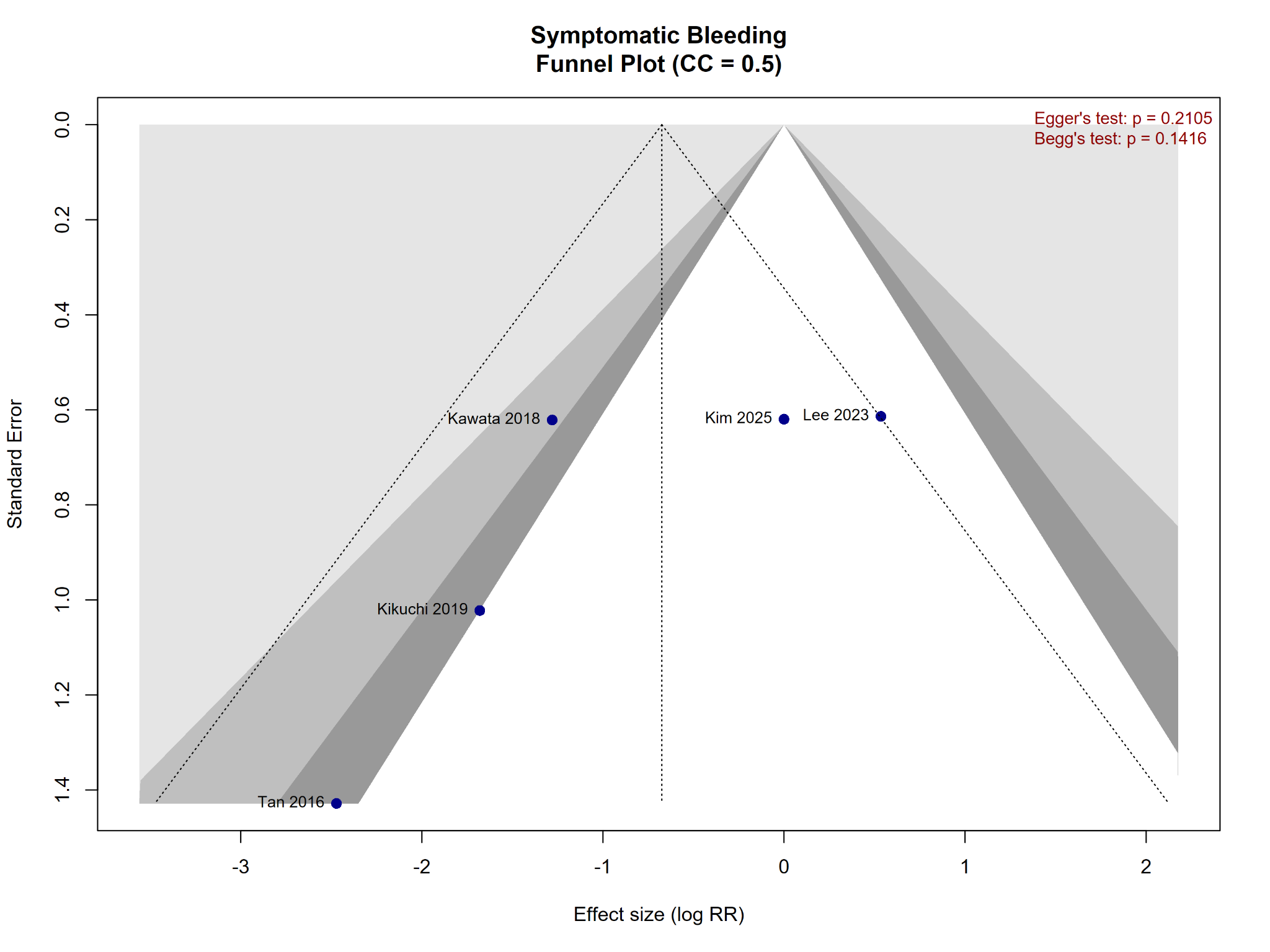


**Figure S15. Perforation - funnel plot (continuity correction = 0.5).**


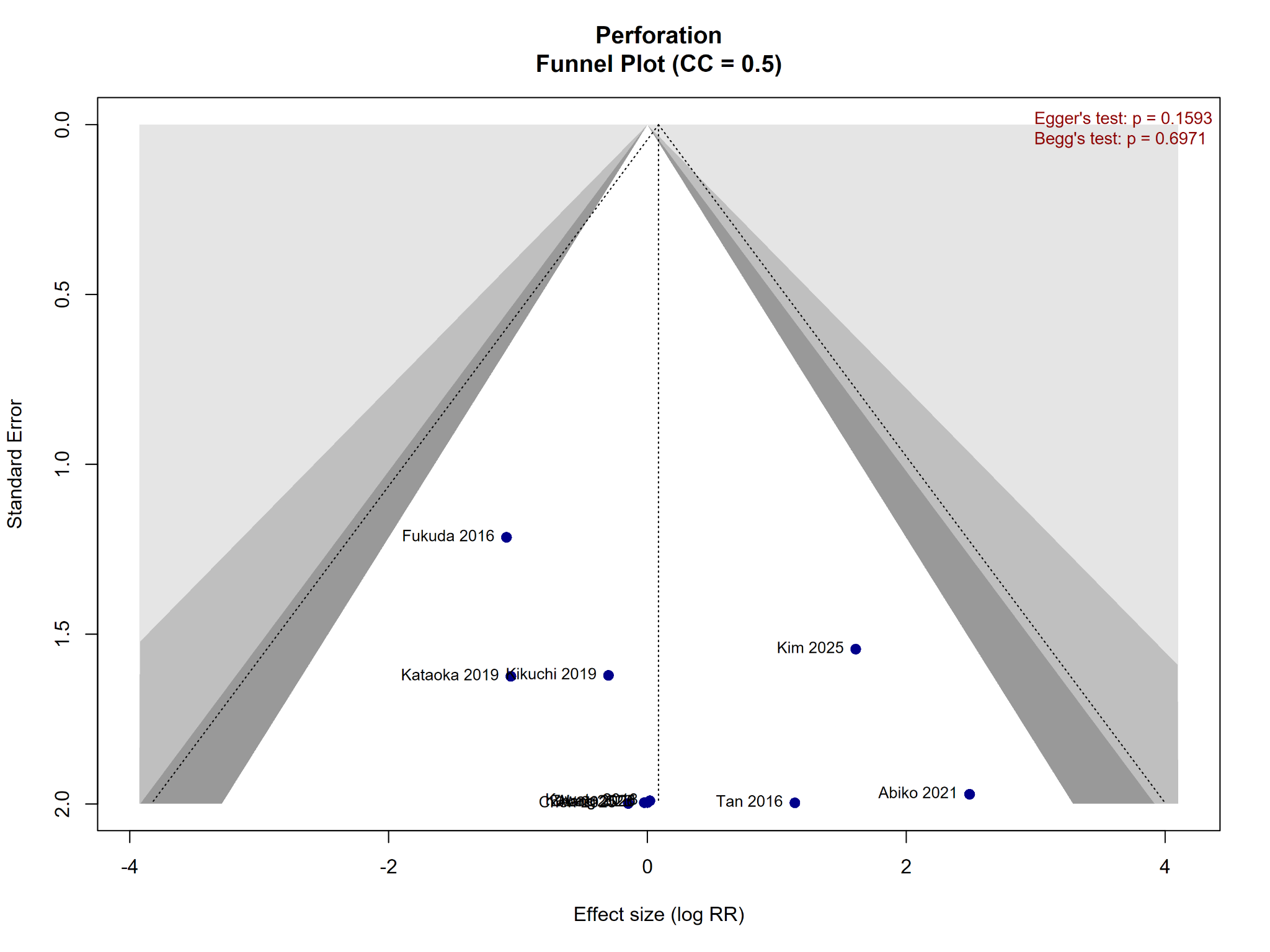

Supplement: Supplementary file 1 — Supplementary Material 1 [file 12029_2026_1502_MOESM1_ESM.docx]
